# ﻿*Australosagola*, a new genus of pselaphine beetles from southern Australia (Coleoptera, Staphylinidae, Pselaphinae, Faronitae) with descriptions of seven new species

**DOI:** 10.3897/zookeys.1245.151556

**Published:** 2025-07-15

**Authors:** Su-Ho Choi, Donald S. Chandler, Jong-Seok Park

**Affiliations:** 1 Department of Biological Sciences and Biotechnology, Chungbuk National University, 1 Chungdae-ro, Seowon-gu, Cheongju-si, Chungbuk 28644, Republic of Korea Chungbuk National University Cheongju-si Republic of Korea; 2 Department of Biological Sciences, University of New Hampshire, Durham, NH 03824, USA University of New Hampshire Durham United States of America

**Keywords:** Ant-loving beetle, Gondwana, new combination, *Sagola*, taxonomy

## Abstract

The new Australian genus, *Australosagola*, **gen. nov.** supertribe Faronitae, is described with seven new species and three new combinations for species previously assigned to *Sagola* Sharp, 1874. This genus includes *Australosagolatasmaniae* (Lea, 1911), **comb. nov.** (type species), *A.rugicornis* (Oke, 1932), **comb. nov.**, *A.helenae* (Oke, 1925), **comb. nov.**, *A.minsangi***sp. nov.**, *A.minhoi***sp. nov.**, *A.jiwooki***sp. nov.**, *A.sunheei***sp. nov.**, *A.doohyungi***sp. nov.**, *A.jungjooni***sp. nov.**, and *A.yongsooni***sp. nov.** A key, illustrations of major diagnostic characters, habitus images, and distribution maps are provided.

## ﻿Introduction

The supertribe Faronitae (Coleoptera: Staphylinidae: Pselaphinae) consists of 30 genera, with 14 of these documented from Australia and New Zealand (Park and Carlton 2014; Park and Chandler 2017; Choi et al. 2019). Four faronite genera [*Sagola*Sharp 1874 (9 spp.), *Logasa* Chandler, 2001 (3 spp.), *Nornalup* Park & Chandler, 2017 (3 spp.), and *Porongurup* Choi, Chandler & Park, 2019 (3 spp.)] have been identified in Australia, and there are more than ten genera with 100 species remaining to be treated.

**Figure 1.**
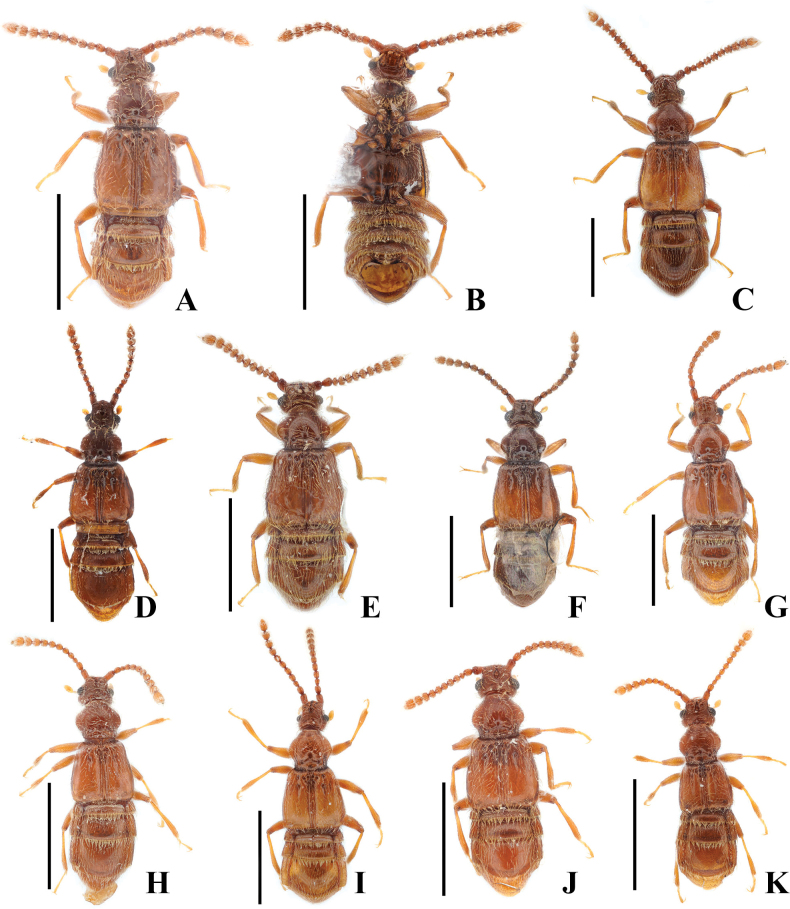
*Australosagola* male habitus photos, all dorsal view except for B, ventral view. **A, B.***A.tasmaniae* comb. nov.; **C.***A.rugicornis* comb. nov.; **D.***A.helenae* comb. nov.; **E.***A.minsangi* sp. nov.; **F.***A.minhoi* sp. nov.; **G.***A.jiwooki* sp. nov.; **H.***A.sunheei* sp. nov.; **I.***A.doohyungi* sp. nov.; **J.***A.jungjooni* sp. nov.; **K.***A.yongsooni* sp. nov. Scale bars: 1.0 mm.

**Figure 2.**
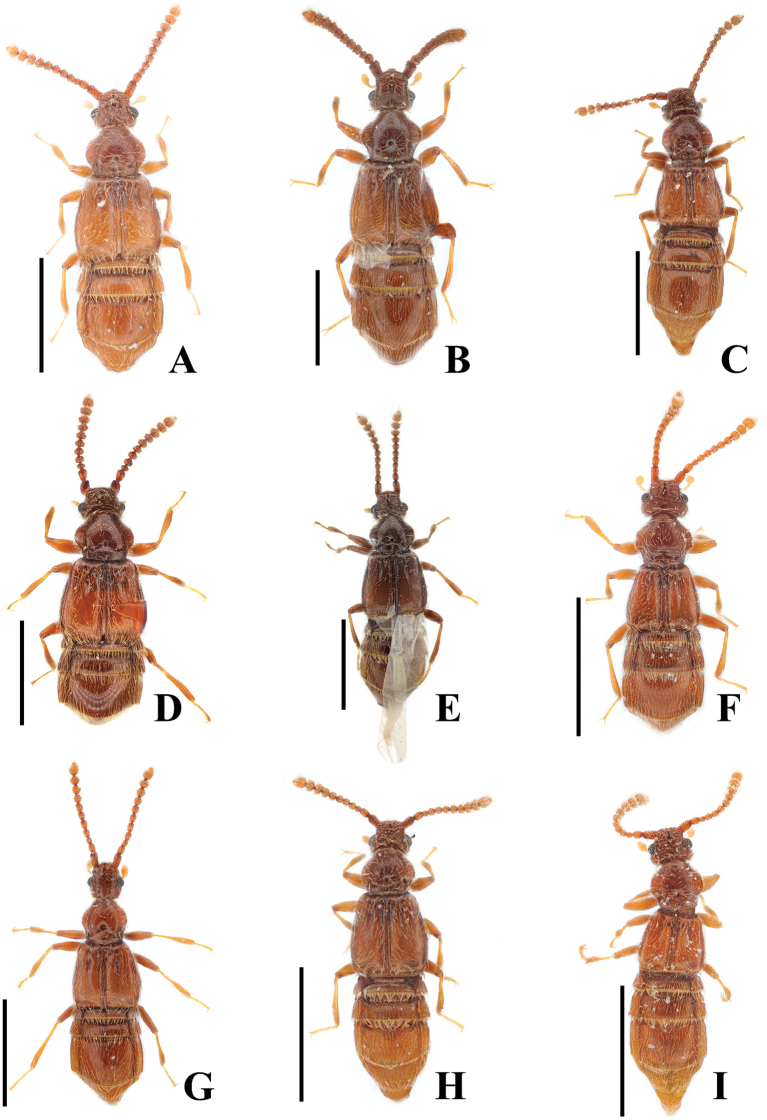
*Australosagola* female habitus photos, dorsal view. **A.***A.tasmaniae* comb. nov.; **B.***A.rugicornis* comb. nov.; **C.***A.helenae* comb. nov.; **D.***A.minsangi* sp. nov.; **E.***A.sunheei* sp. nov.; **F.***A.minhoi* sp. nov.; **G.***A.doohyungi* sp. nov.; **H.***A.jungjooni* sp. nov.; **I.***A.yongsooni* sp. nov. Scale bars: 1.0 mm.

**Figure 3.**
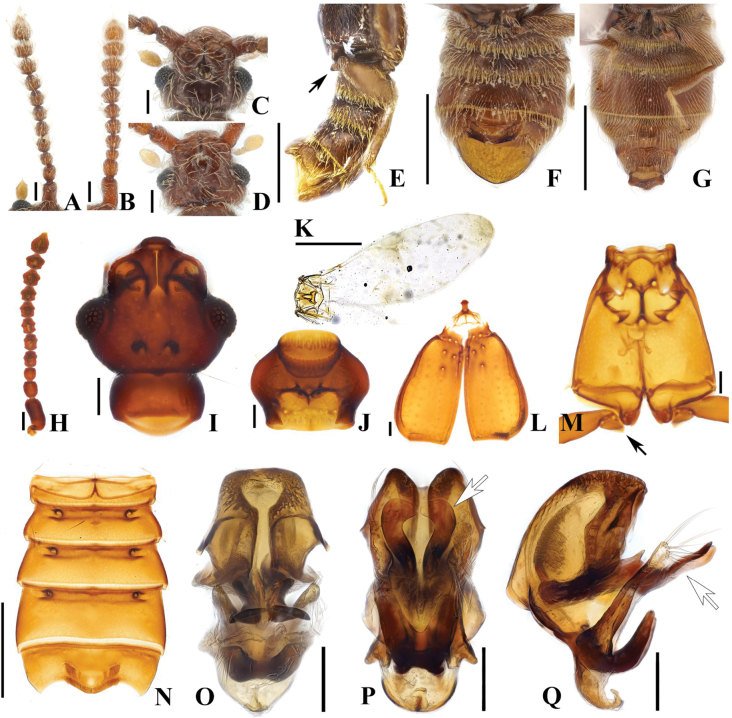
*Australosagolatasmaniae* comb. nov. (**A, C, E–F, H–Q**) male; (**B, D, G**) female. **A, B.** Antennae; **C, D.** Head, dorsal view; **E.** Abdomen, lateral view; **F, G.** Abdomen, ventral view; **H.** Antenna; **I.** Head, dorsal view; **J.** Prothorax, ventral view; **K.** Hind wing; **L.** Elytra, dorsal view; **M.** Meso-metathorax; **N.** Abdomen, ventral view; **O.** Aedeagus, ventral view; **P.** Aedeagus, basoventral view; **Q.** Aedeagus, lateral view. Scale bars: 0.1 mm (**A–D, H–J, L, M, O–Q**); 0.5 mm (**E–G, N**); 1 mm (**K**).

**Figure 4.**
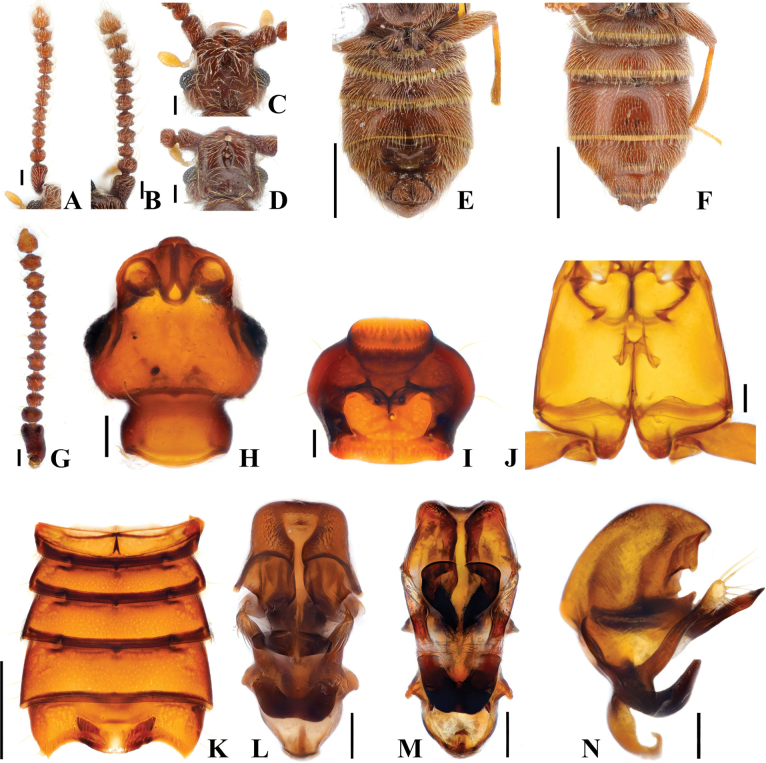
*Australosagolarugicornis* comb. nov. (**A, C, E, G–N**) male; (**B, D, F**) female. **A, B.** Antennae; **C, D.** Head, dorsal view; **E, F.** Abdomen, ventral view; **G.** Antenna; **H.** Head, dorsal view; **I.** Prothorax, ventral view; **J.** Metathorax, ventral view; **K.** Abdomen, ventral view; **L.** Aedeagus, ventral view; **M.** Aedeagus, basoventral view; **N.** Aedeagus, lateral view. Scale bars: 0.1 mm (**A–D, G–J, L–N**); 0.5 mm (**E, F, K**).

**Figure 5.**
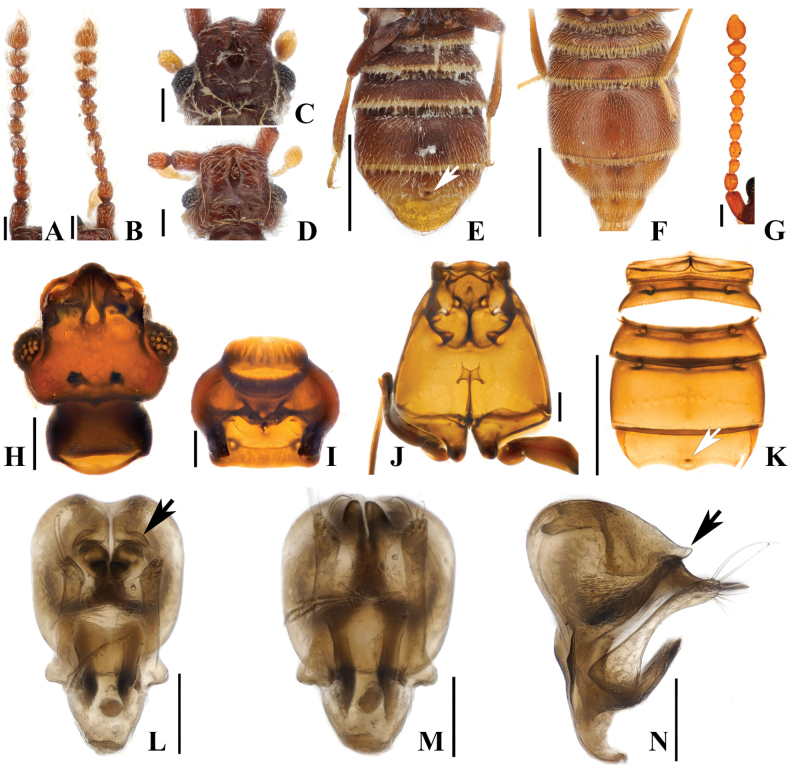
*Australosagolahelenae* comb. nov. **(A, C, E, G–N)** male; **(B, D, F)** female. **A, B.** Antennae; **C, D.** Head, dorsal view; **E, F.** Abdomen, ventral view; **G.** Antenna; **H.** Head, dorsal view; **I.** Prothorax, ventral view; **J.** Meso-metathorax, ventral view; **K.** Abdomen, ventral view; **L.** Aedeagus, ventral view; **M.** Aedeagus, basoventral view; **N.** Aedeagus, lateral view. Scale bars: 0.1 mm (**A–D, G–J, L–N**); 0.5 mm (**E, F, K**).

**Figure 6.**
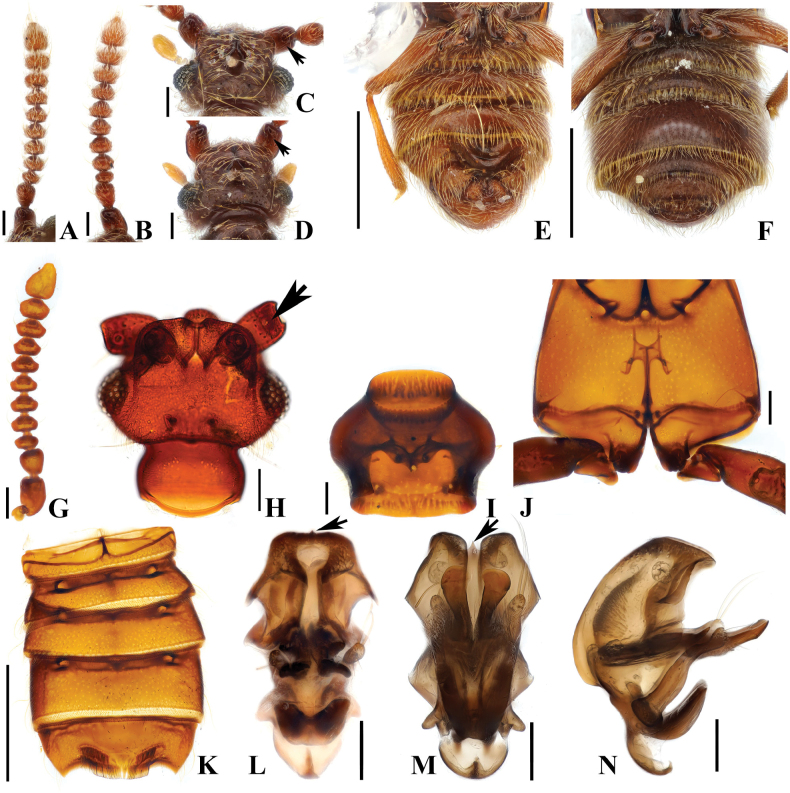
*Australosagolaminsangi* sp. nov. (**A, C, E, G–N**) male; (**B, D, F**) female. **A, B.** Antennae; **C, D.** Head, dorsal view; **E, F.** Abdomen, ventral view; **G.** Antenna; **H.** Head, dorsal view; **I.** Prothorax, ventral view; **J.** Meso-metathorax, ventral view; **K.** Abdomen, ventral view; **L.** Aedeagus, ventral view; **M.** Aedeagus, basoventral view; **N.** Aedeagus, lateral view. Scale bars: 0.1 mm (**A–D, G–J, L–N**); 0.5 mm (**E, F, K**).

**Figure 7.**
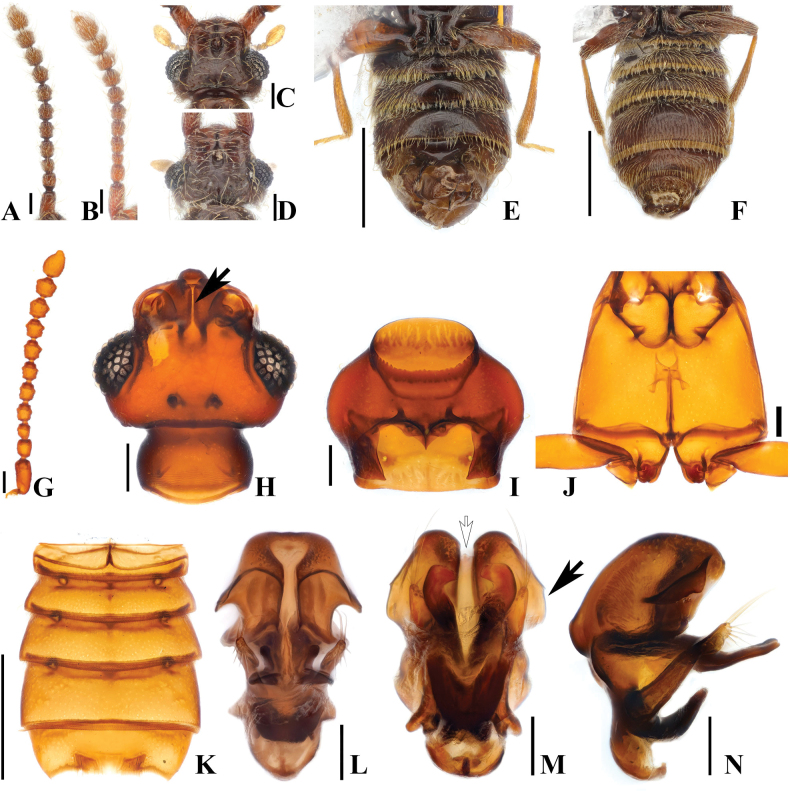
*Australosagolaminhoi* sp. nov. (**A, C, E, G–N**) male; (**B, D, F**) female. **A, B.** Antennae; **C, D.** Head, dorsal view; **E, F.** Abdomen, ventral view; **G.** Antenna; **H.** Head, dorsal view; **I.** Prothorax, ventral view; **J.** Metathorax, ventral view; **K.** Abdomen, ventral view; **L.** Aedeagus, ventral view; **M.** Aedeagus, basoventral view; **N.** Aedeagus, lateral view. Scale bars: 0.1 mm (**A–D, G–J, L–N**); 0.5 mm (**E, F, K**).

**Figure 8.**
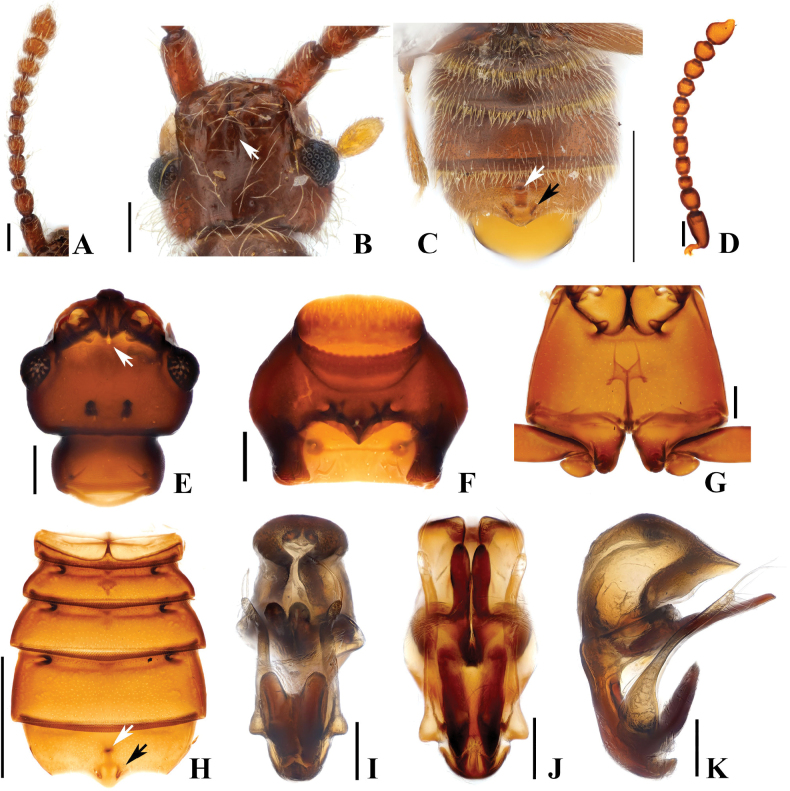
*Australosagolajiwooki* sp. nov. male. **A.** Antenna; **B.** Head, dorsal view; **C.** Abdomen, ventral view; **D.** Antenna; **E.** Head, dorsal view; **F.** Prothorax, ventral view; **G.** Metathorax, ventral view; **H.** Abdomen, ventral view; **I.** Aedeagus, ventral view; **J.** Aedeagus, basoventral view; **K.** Aedeagus, lateral view. Scale bars: 0.1 mm (**A, B, D–G, I–K**); 0.5 mm (**C, H**).

**Figure 9.**
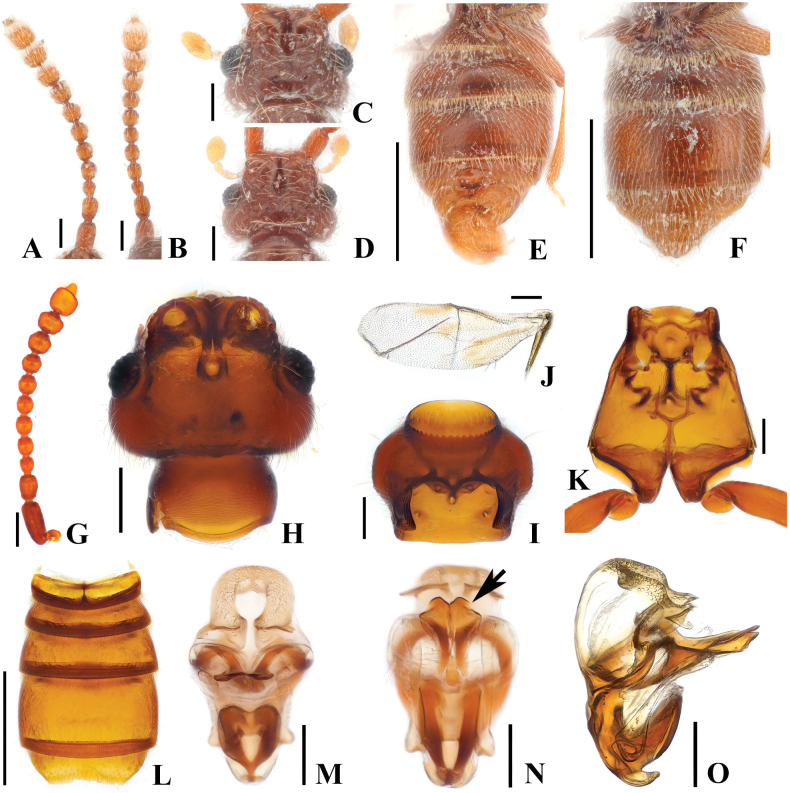
*Australosagolasunheei* sp. nov. (**A, C, E, M–O**) male; (**B, D, F, G–L**) female. **A, B.** Antennae; **C, D.** Head, dorsal view; **E, F.** Abdomen, ventral view; **G.** Antenna; **H.** Head, dorsal view; **I.** Prothorax, ventral view; **J.** Hind wing; **K.** Meso-metathorax, ventral view; **L.** Abdomen, ventral view; **M.** Aedeagus, ventral view; **N.** Aedeagus, basoventral view; **O.** Aedeagus, lateral view. Scale bars: 0.1 mm (**A–D, G–K, M–O**); 0.5 mm (**E, F, L**).

**Figure 10.**
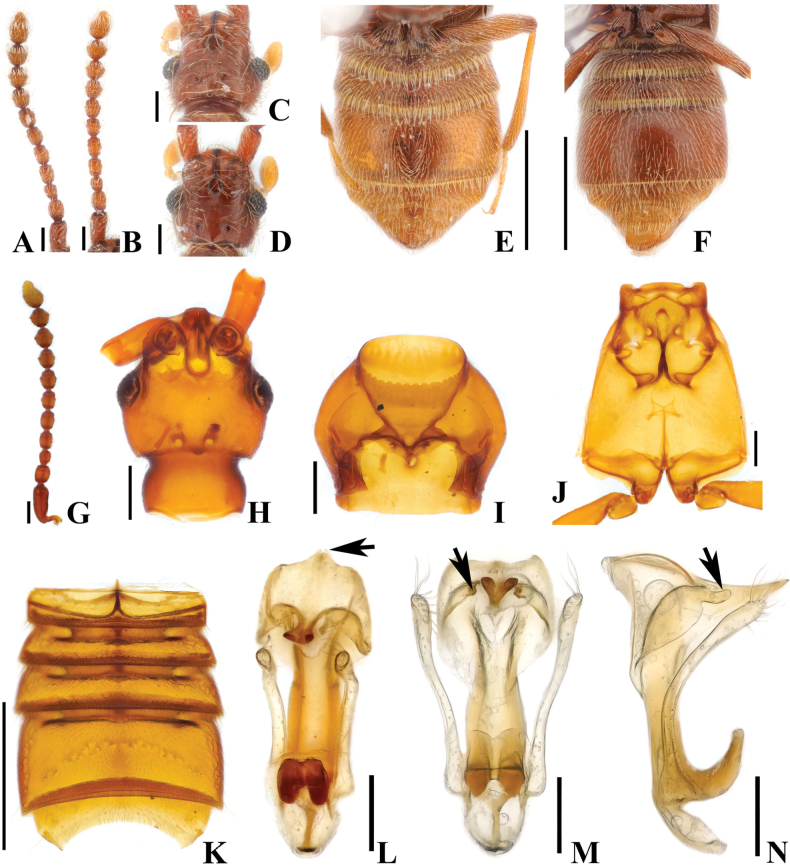
*Australosagoladoohyungi* sp. nov. (**A, C, E, G–N**) male; (**B, D, F**) female. **A, B.** Antennae; **C, D.** Head, dorsal view; **E, F.** Abdomen, ventral view; **G.** Antenna; **H.** Head, dorsal view; **I.** Prothorax, ventral view; **J.** Meso-metathorax, ventral view; **K.** Abdomen, ventral view; **L.** Aedeagus, ventral view; **M.** Aedeagus, basoventral view; **N.** Aedeagus, lateral view. Scale bars: 0.1 mm (**A–D, G–J, L–N**); 0.5 mm (**E, F, K**).

**Figure 11.**
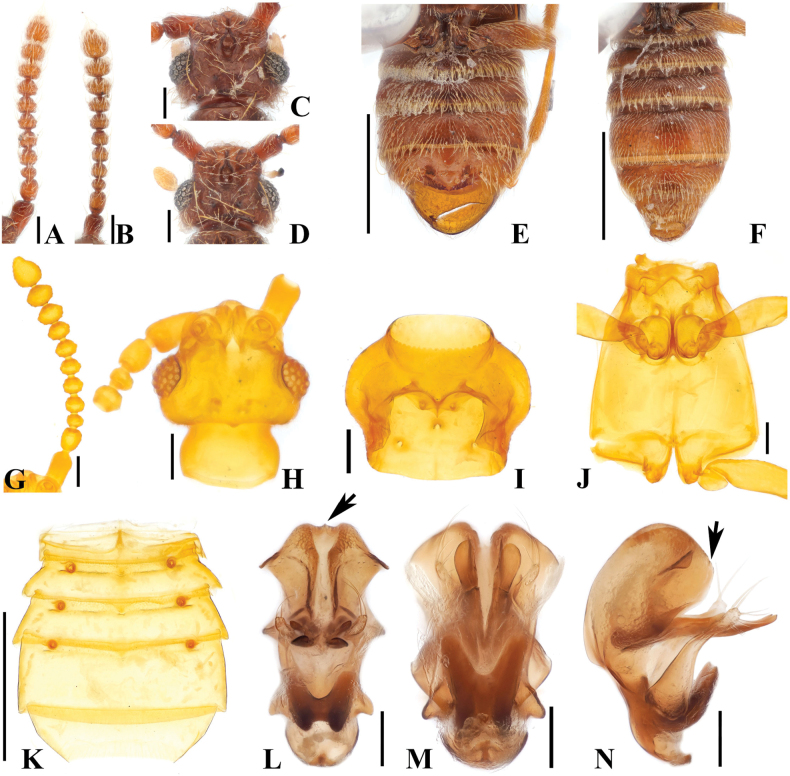
*Australosagolajungjooni* sp. nov. (**A, C, E, L–N**) male; (**B, D, F–K**) female. **A, B.** Antennae; **C, D.** Head, dorsal view; **E, F.** Abdomen, ventral view; **G.** Antenna; **H.** Head, dorsal view; **I.** Prothorax, ventral view; **J.** Meso-metathorax, ventral view; **K.** Abdomen, ventral view; **L.** Aedeagus, ventral view; **M.** Aedeagus, basoventral view; **N.** Aedeagus, lateral view. Scale bars: 0.1 mm (**A–D, G–J, L–N**); 0.5 mm (**E, F, K**).

**Figure 12.**
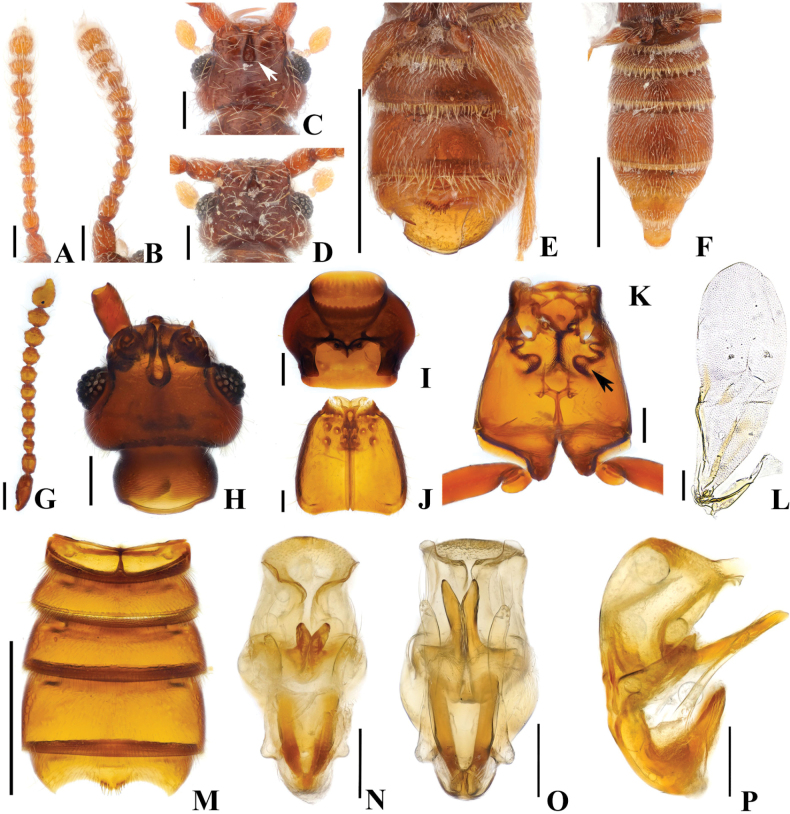
*Australosagolayongsooni* sp. nov. (**A, C, E, G–P**) male (**B, D, F**) female. **A, B.** Antennae; **C, D.** Head, dorsal view; **E, F.** Abdomen, ventral view; **G.** Antenna; **H.** Head, dorsal view; **I.** Prothorax, ventral view; **J.** Elytra, dorsal view, **K.** Meso-metathorax, ventral view; **L.** Hind wing; **M.** Abdomen, ventral view; **N.** Aedeagus, ventral view; **O.** Aedeagus, basoventral view; **P.** Aedeagus, lateral view. Scale bars: 0.1 mm (**A–D, G–L, N–P**); 0.5 mm (**E, F, M**).

**Figure 13.**
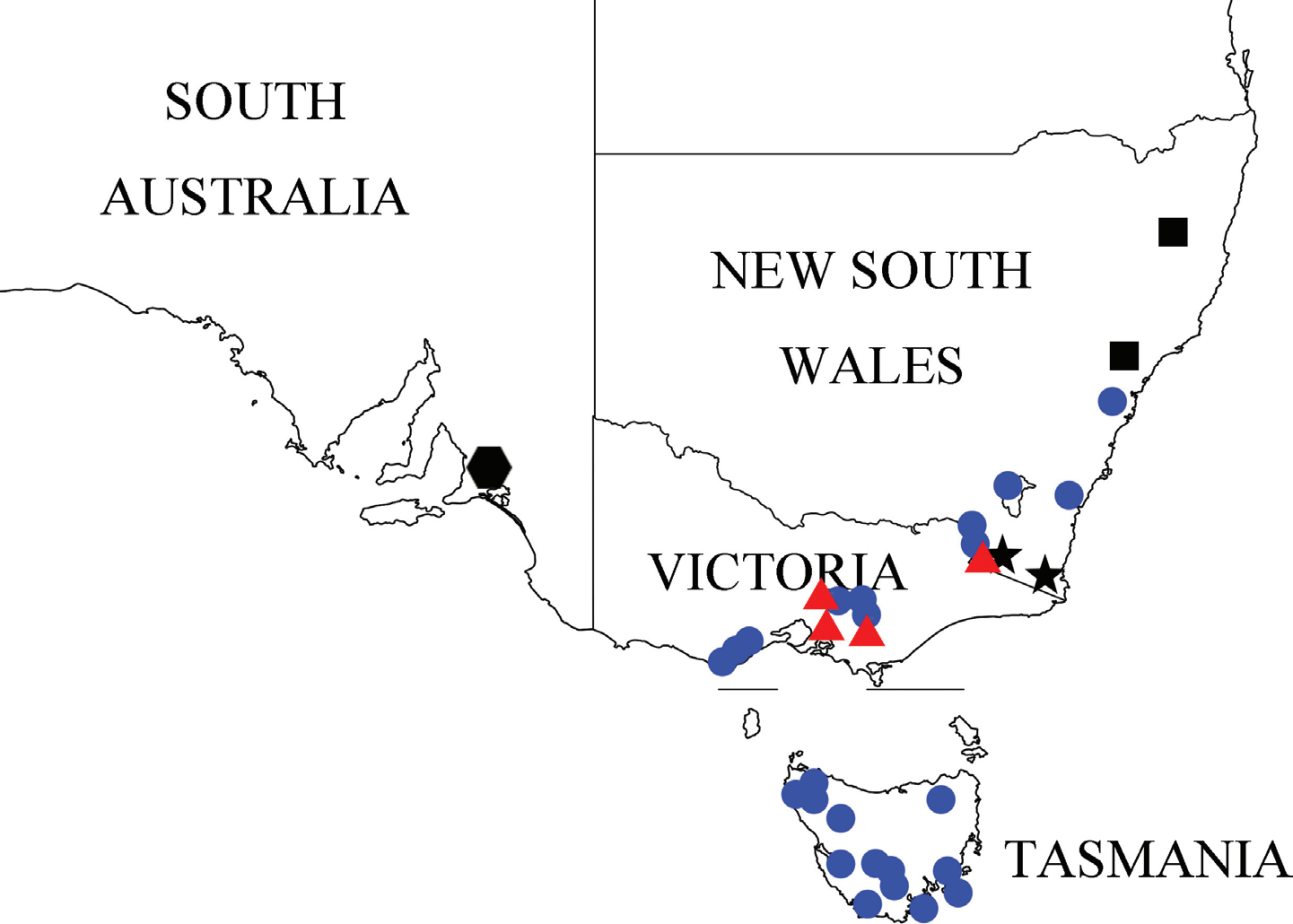
Collection localities of *Australosagola* species. *A.tasmaniae* comb. nov.: blue circles; *A.rugicornis* comb. nov.: red triangles; *A.minsangi* sp. nov.: black squares; *A.sunheei* sp. nov.: black hexagon; *A.jungjooni* sp. nov.: black stars.

**Figure 14.**
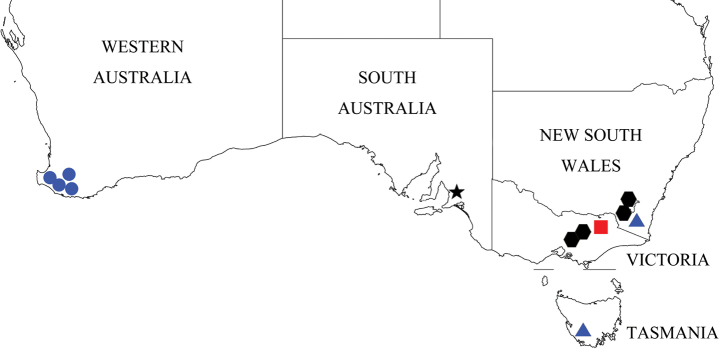
Collection localities of *Australosagola* species. *A.helenae* comb. nov.: black hexagons; *A.jiwooki* sp. nov.: red square; *A.minhoi* sp. nov.: blue triangles; *A.yongsooni* sp. nov.: black star; *A.doohyungi* sp. nov.: blue circles.

During examination of faronite specimens from museums and institutional collections, we identified and separated 366 specimens in ten species, including 217 individuals of three Australian *Sagola* species [*S.rugicornis* Oke, *S.tasmaniae* Lea, and *S.helenae* Oke] (Figs 1A–D, 2A–C). These species (Figs 1–12) possess diagnostic characters not shared with any currently recognized faronite genera, assigned to a new genus. This new genus is distributed exclusively in the southern half of Australia (New South Wales, Victoria, Tasmania, South Australia, and southern part of West Australia; Figs 13, 14).

## ﻿Materials and methods

A total of 366 specimens (323 dried, 41 slide-mounted, 2 mounted in micro vial) were examined from the following collections:

**ANIC**Australian National Insect Collection, Canberra, Australian Capital Territory, Australia;

**FMNH**Field Museum of Natural History, Chicago, Illinois, USA;

**MVMA** National Museum of Victoria, Melbourne, Victoria, Australia;

**SAMA**South Australian Museum, Adelaide, South Australia, Australia;

**UNHC**University of New Hampshire Insect Collection, Durham, New Hampshire, USA.

Forty-one specimens were fully dissected to observe their internal and external characters. Permanent microscopic slide mounts were prepared using the techniques described by Hanley and Ashe (2003). The foveal system adheres to the terminology established by Chandler (2001) and Lawrence et al. (2011). Abdominal tergites and sternites are identified by their position based on a sequence of visibility (Arabic numbers) and morphological position (Roman numerals). Parts of the paired appendages are referred to as singular; paired structures of the body are treated as plural. For convenience, the orientation of the genitalic structures is described based on their position in the figures rather than on their true morphological position. To clarify the morphology, we have included two photos of the male antennae, one detached and one intact. Geographical coordinates are presented in Degree Decimal and Minutes (DDM) format. The holotypes are deposited in the collections of the ANIC, SAMA, and MVMA. Specimens were observed using a Leica S9E. Images were generated by a Sony ILCE-7RM3 camera with 10X / 20X Mitutoyo Plan Apo Objective, and stacked using Zerene Stacker version 1.04 and Helicon Focus version 8.2.2. Specimen label data for the holotypes are transcribed verbatim. The ‘/’ was used to indicate a line break, and ‘//’ was used to indicate a label break in the label data of holotypes. Data for paratypes are standardized for consistency. The map of Australia was based on an image from SimpleMappr (Shorthouse 2010) and was subsequently modified to indicate the locality of specimens.

Adults were typically found by sampling leaf litter and rotten wood in wet and dry sclerophyll forests, with some taken in cool or warm temperate rainforests and *Eucalyptus* woodlands. Adults were most frequently collected by sifting in leaf/wood litter and placing Berlese funnels, and spraying pyrethrin on logs, but some were also taken by use of flight intercept traps.

## ﻿Systematics

**Family Staphylinidae Latreille, 1802**

**Subfamily Pselaphinae Latreille, 1802**

**Supertribe Faronitae Reitter, 1882**

### ﻿Genus Australosagola gen. nov.

Taxon classificationAnimaliaColeopteraStaphylinidae

B5F36D0D-35B6-5C9A-8074-F9E1CD040D08

https://zoobank.org/224545F1-ED61-4EF1-BFE2-6A21D982A856

#### Type species.

*Sagolatasmaniae* Lea, 1911, here designated.

#### Diagnosis.

Members of this genus are separated from other faronite genera by the following combination of characters: prominent rostrum of head with narrow, elongate median frontal sulcus terminating posteriorly at frontal fovea, median sulcus impressed at midpoint of head around frontal fovea (Fig. 3C, D, I); pronotum with isolated median antebasal fovea, lateral antebasal foveae, and inner basolateral foveae; prosternum with median procoxal fovea and lateral procoxal foveae (Fig. 3J); elytra with two subbasal elytral foveae, three basal elytral foveae (1 at base of sutural stria), discal elytral foveae with short discal striae, and additional fovea present in sutural striae (Fig. 3L); mesoventrite with lateral mesoventral foveae, lateral mesocoxal foveae, and promesocoxal foveae; metaventrite with median metaventral fovea, and lateral metaventral foveae (Fig. 3M); abdominal tergite 2 (V) ~2/3 length of 3 (VI) (Figs 1, 2). Species only known from southern half of Australia (Figs 13, 14).

#### Description.

1.8–3.5 mm (Figs 1, 2). Body brown to dark brown. ***Head*.** Head broader than long to as long as wide, widest across eyes for both sexes, rostrum longitudinally divided by narrow median frontal sulcus (Fig. 3C, D, I). Antennomeres modified in some species. ***Thorax*.** Prothorax broader than long, widest at midpoint (Fig. 3J). Meso-metathorax trapezoidal in ventral view, widest at posterior margin (Fig. 3M). Males of some species have metatrochanters with angulate ventral margins (Fig. 3E, F, M, black arrow). ***Abdomen*.** Most male specimens have posteriorly directed large rows of large spinoid setae near posterior margin of sternite 5 (VII) (Fig. 3F, N). ***Genitalia*.** Length 0.32–0.57 mm. All members in this genus with symmetrical aedeagus with expanded bulbous form of median lobe; with paired curved projections at base of median lobe best visible in lateral view (Fig. 3O–Q), elongated parameres with apices bearing long, sparse setae. Every species except *A.doohyungi* sp. nov., has elongate projection from near midpoint of median lobe (Fig. 3P, white arrow, Q, white arrow).

#### Etymology.

*Australosagola* gen. nov. is named for its endemic locality (Australia) and its similarity to the genus *Sagola*. Gender feminine.

#### Distribution.

Southern part of Western Australia, South Australia, New South Wales, Victoria, Tasmania, and the Australian Capital Territory (Figs 13, 14).

#### Comments on secondary sexual characters.

Except for *A.doohyungi* sp. nov., the abdomen of male specimens is medially impressed on venter for either sternites 4 or 5 (VI–VII) or for both (Figs 3F, N, 10E, K). Most species, except for *A.helenae* comb. nov., *A.jiwooki* sp. nov., *A.sunheei* sp. nov., and *A.yongsooni* sp. nov. have the metatrochanters of male specimens with angulate ventral margins (Fig. 3E, F, M).

#### Comments on related taxa.

Both *Australosagola* gen. nov. and *Porongurup* have an almost identical foveal pattern as well as an elongate abdominal tergite 3 (VI). On this basis *Australosagola* is close to the genus *Porongurup*. However, species of *Porongurup* [*P.clarkei* Choi, Chandler & Park, 2019, *P.tenuis* Choi, Chandler & Park, 2019, and *P.angulatus* Choi, Chandler & Park, 2019] have a small frontal fovea that is not more broadly impressed lateral and posterior to the frontal rostrum. Also, the pronotum of members of *Australosagola* is broader than *Porongurup*. The aedeagus of *Australosagola* is bizarrely and consistently different because it is symmetrical, which is very uncommon in Faronitae (in which at least the median lobe is usually asymmetrical). The phallobase is very short, approximately as wide as long, the parameres are narrow and elongate, and the median lobe is almost grotesquely inflated with thick curved spines originating at the base and longer, more complex spines arise from near the middle. In *Australosagola* gen. nov. the frontal rostrum is longitudinally divided by a median sulcus that typically has the margins contiguous, and the head is broadly impressed around the frontal fovea, and the elytra with two subbasal elytral foveae, three basal elytral foveae (1 being fovea at base of sutural stria), discal elytral foveae with short discal striae, and single fovea in sutural striae.

Little can be said about the relationship of *Australosagola* to the other groups present in Australia as at this time a revision of the entire fauna has only started. Of those other genera that are described, *Logasa* Chandler, 2001 has the frontal rostrum medially fused and lacks a frontal sulcus, *Nornalup* Park & Chandler, 2017 has the ventral surface of the head swollen, a broad frontal sulcus, and lacks a median metaventral fovea, while the remaining diverse species are all tentatively left in *Sagola* Sharp sensu lato.

### ﻿Key to males of the species of *Australosagola* gen. nov.

**Table:** 

1	Abdominal sternites 4–5 (VI–VII) medially impressed (Fig. 4E)	**2**
–	Only abdominal sternite 5 (VII) medially impressed (Fig. 8C)	**5**
–	Abdominal sternites 4–5 (VI–VII) broadly convex, neither medially impressed (Fig. 10E)	***A.doohyungi* sp. nov.**
2(1)	Minimum width between eyes ~1/2 head width in dorsal view (Fig. 7C, H)	***A.minhoi* sp. nov.**
–	Minimum width between eyes ~2/3 head width in dorsal view (Fig. 3C, H)	**3**
3(2)	Antennomeres 4–9 strongly constricted at middle (Fig. 4A, G)	***A.rugicornis* comb. nov.**
–	Antennomeres 3–9 strongly transverse, disc-like (Fig. 6A, G)	***A.minsangi* sp. nov.**
–	Antennomeres 3–9 lacking distinct modifications, cylindrical to globular	**4**
4(3)	Antennomere 3 transverse, short (Fig. 11A, G)	***A.jungjooni* sp. nov.**
–	Antennomere 3 quadrate, approx. as long as wide (Fig. 3A, H)	***A.tasmaniae* comb. nov.**
5(1)	Abdominal sternite 5 (VII) with two small clusters or line of thick setae at apex of median apical angulation (Fig. 3F, N)	**6**
–	Abdominal sternite 5 (VII) lacking paired clusters or line of thick setae on median apical angulation, with small preapical median tubercle (Fig. 5E, white arrow, K, white arrow)	***A.helenae* comb. nov.**
6(5)	Frontal fovea large, at least twice as wide as frontal sulcus (Fig. 12C, white arrow, H)	**7**
–	Frontal fovea small, barely wider than frontal sulcus (Fig. 8B, white arrow, E, white arrow)	***A.jiwooki* sp. nov.**
7(6)	Frontal sulcus with lateral margins widening posteriorly, in form of teardrop (Fig. 12C, white arrow, H)	***A.yongsooni* sp. nov.**
–	Frontal sulcus with lateral margins linear (Fig. 9C, H)	***A.sunheei* sp. nov.**

### ﻿Species descriptions

### ﻿ Australosagola tasmaniae

Taxon classificationAnimaliaColeopteraStaphylinidae

(Lea, 1911) comb. nov.

16E17D79-1BF1-5ABC-85FC-AC26A06C510A

Figs 1A, B , 2A , 3 , 13

Sagola tasmaniae Lea, 1911: 693, pl. XXI, fig. 1. Type localities: New Norfolk, and Mt. Wellington, Tasmania. Lectotype male (SAMA).

#### Type material.

***Lectotype*. Australia: Tasmania**: • ♂ (SAMA), “1415.6 / Sagola tasmaniae Lea Tasmania // tasmaniae / Lea, Type / Mt Wellington // LECTOTYPE first ♂ on left / Sagola tasmaniae Lea / other 2 ♂♂ PARALECTOTYPES / det. DSChandler, 87 // SAMA Database No. 25-036493 // SA museum / Duplicate specimens in alcohol.” ***Paralectotypes*** (*n* = 2; 2 ♂♂). • 2 ♂♂ (mounted with lectotype; SAMA), same data as lectotype.

#### Other material examined

(*n* = 164; 110 ♂♂, 54 ♀♀). See Suppl. material 1.

#### Diagnosis.

This species can be distinguished by following characters: antennomeres 4–10 slightly constricted at middle (Fig. 3A, B, H); slightly opened rostrum (Fig. 3I); angulate ventral margin of metatrochanter (Fig. 3E, M); concave abdominal sternites 4 and 5 (VI–VII) in male; distinct setate margin of abdominal sternite 5 (VII) (Fig. 3F, N).

#### Male description.

Length. 2.5–2.8 mm. Body reddish-brown. ***Head*.** Head in dorsal view with large impression at midpoint of head at area of frontal fovea. Vertexal foveae well-developed (Fig. 3I). Antennomere 1 cylindrical, longer than wide; 2 subquadrate and as long as wide; 3 smallest, subconical and as long as wide; 4–6 longer than wide and slightly constricted at middle; 7 and 8 as long as wide and slightly constricted at middle; 9 and 10 transverse and slightly constricted at middle (Fig. 3A, H). ***Thorax*.** Prothorax slightly broader than long for both sexes, widest at midpoint (Fig. 3J). Elytra with two subbasal elytral foveae, three basal elytral foveae (1 being fovea at base of sutural stria), discal elytral foveae with short discal striae, and fovea in sutural striae (Fig. 3L). Hind wings fully developed (Fig. 3K). Metatrochanter angulate on ventral margin, distinct in lateral view [Fig. 3E (black arrow), F, M (black arrow)]. ***Abdomen*.** Median part of abdominal sternites 4 and 5 (VI–VII) both largely impressed. Abdominal sternite 5 (VII) with lateral preapical rows and apical row of thick setae on medial apical projection (Fig. 3F, N). ***Genitalia*.** Length 0.37 mm, aedeagus symmetrical, pair of elongate dentate projections near midpoint of median lobe, curved inward at apex in basoventral view (Fig. 3P, white arrow), slightly narrower in lateral view, hook-shaped projection on basal part of median lobe best visible in lateral view, projection forming short broad U in ventral view, phallobase with lateral margins evenly rounded in basoventral view, flat and strongly curved in lateral view (Fig. 3O–Q).

#### Female sexual characters.

Metatrochanter smoothly convex. Abdominal sternites 4 and 5 (VI–VII) broadly convex, lacking thick setae at apex of abdo­minal sternite 5 (VII) (Fig. 3G).

#### Comments.

*Australosagolatasmaniae* comb. nov. resembles to *A.rugicornis* comb. nov., and *A.minhoi* sp. nov., but it can be easily separated from *A.rugicornis* by the features of the antennomeres, which are greatly constricted at the middle in *A.rugicornis* (Fig. 4A, B, G). And it differs from *A.minhoi* in the size of the eyes, with the minimum width between eyes being ~1/2 the head width in dorsal view (Fig. 7C, D, H). Based on the weakly constricted antennomers of the figure of *S.rugicornis* in Chandler (2001: fig. 32), the specimen illustrated is a specimen of *S.tasmaniae*. See Oke (1932: 150, fig. 3) for a figure of the antennae, and the figure of *S.tasmaniae* in Lea (1911: pl. XXI, fig. 1), which both show unmodified antennomeres.

#### Distribution.

Found in the Australian Capital Territory, Tasmania, and Victoria (Fig. 13, blue circles).

#### Habitat.

Specimens of this species were collected using flight intercept traps (F.I.T.), yellow pan traps, by spraying pyrethrin on *Anthospermum* and *Nothofagus* tree trunks, by sifting leaf litter in *Eucalyptus* forests, or by use of emergence traps. Found in rainforests and wet sclerophyll forests dominated by *Nothofaguscunninghami* or *Eucalyptus* and *Acacia* species.

### ﻿ Australosagola rugicornis

Taxon classificationAnimaliaColeopteraStaphylinidae

(Oke, 1932) comb. nov.

2542BC8C-F5C8-5B25-9EBA-37B5D7CADDF6

Figs 1C , 2B , 4 , 13

Sagola rugicornis Oke, 1932: 149, fig. 3. Type locality: Warburton, Mt. Donna Buang, Victoria. Holotype male (MVMA). Chandler 2001: 52, figs 32, 196, both A.tasmaniae.

#### Type material.

***Holotype*. Australia: Victoria**: • ♂ (MVMA), “WARBURTON, Vic. / 18 . 2 . 81 / C. OKE. 4088’ // 1081 Type ♂ // MUS. VIC. ENTO 2015-1L”.

#### Other material examined

(*n* = 23; 9 ♂♂, 14 ♀♀). See Suppl. material 1.

#### Diagnosis.

Adult specimens of *A.rugicornis* comb. nov. can be separated from other species in *Australosagola* by the distinct form of antennomeres with antennomeres 4–8 being strongly constricted at their middle (Fig. 4A, B, G).

#### Male description.

Length 2.8–3.3 mm. Body reddish-brown. ***Head*.** Head in dorsal view with large impression at base of frontal rostrum around frontal fovea. Vertexal foveae well-developed (Fig. 4C, H). Antennomere 1 subconical and longer than wide, slightly curved; 2 rounded and transverse; 3 smallest and subconical, slightly constricted at middle; 4–8 distinctly constricted at middle and as long as wide; 9 and 10 distinctly constricted at middle and transverse (Fig. 4A, G). ***Thorax*.** Prothorax slightly broader than long, widest at midpoint (Fig. 4I). Elytra with two subbasal elytral foveae, three basal elytral foveae (1 being fovea at base of sutural stria), discal elytral foveae with short discal striae, and fovea in sutural striae. Hind wings fully developed. Metatrochanter with angulate ventral margin (Fig. 4E, J). ***Abdomen*.** Abdominal sternites 4 and 5 (VI–VII) both strongly impressed medially (Fig. 4E). Abdominal sternite 5 (VII) with large lateral preapical and apical rows of setae at middle (Fig. 4E, K). ***Genitalia*.** Length 0.57 mm, aedeagus symmetrical, with pair of long dentate projections near midpoint of median lobe, projections curved medially at apices in basoventral view (Fig. 4M) and acutely pointed in lateral view (Fig. 4N). Projections at basal part of median lobe strongly curved apically (Fig. 4L, M).

#### Female sexual characters.

Antennomere 1 stout, subconical and longer than wide; 2 transverse and subquadrate; 3 smallest, subconical and slightly transverse; 4 and 5 distinctly constricted at middle and as long as wide; 6–9 distinctly constricted at middle and transverse; 10 slightly constricted at middle and transverse (Fig. 4B). Metatrochanter with ventral margin convex (Fig. 4F). Abdominal sternites 4 and 5 (VI–VII) convex, lacking thick setae at apex of abdominal sternite 5 (VII; Fig. 4F).

#### Comments.

The aedeagi of *A.rugicornis* and *A.tasmaniae* comb. nov. are similar, but the aedeagus of *A.rugicornis* is notably larger (length of *A.tasmaniae* aedeagus 0.37 mm, for *A.rugicornis* it is 0.57 mm) (Figs 3O–Q, 4L–N), and the projections from the midpoint of the median lobe are longer and differently formed for *A.tasmaniae* (Figs 3P (white arrow), Q (white arrow), 4M, N). The temples of *A.tasmaniae* are rounded, whereas those of *A.rugicornis* are angular (Figs 3I, 4H). Additionally, the setal cluster on abdominal sternite 5 (VII) is medially open in *A.rugicornis*, whereas it is complete in *A.tasmaniae* (Figs 3N, 4K).

#### Distribution.

Victoria and New South Wales (Fig. 13, red triangles).

#### Habitat.

Specimens of this species were collected using flight intercept traps (F.I.T.), by sifting leaf, log, and forest floor litter, and found in debris under a rock in *Eucalyptus* forests, or by spraying pyrethrin on *Eucalyptus* logs. Taken primarily in *Nothofaguscunninghami* and *Eucalyptusregnans* wet sclerophyll forests.

### ﻿ Australosagola helenae

Taxon classificationAnimaliaColeopteraStaphylinidae

(Oke, 1925) comb. nov.

F59EED14-4715-5D0D-8616-5A59FCA16FEF

Figs 1D , 2C , 5 , 14

Sagola helenae Oke, 1925: 7. Type locality: Evelyn, Victoria. Holotype male (MVMA). Chandler 2001: 52.

#### Type material.

***Holotype*. Australia: Australian Capital Territory**: • ♂ (MVMA), “Evelyn.V. / s.6.22 / C. Oke // Sagola / helenae / ♂ Oke // 1059 / Type ♂ // MUS VIC. / ENTO 2015-1L”.

#### Other material examined

(*n* = 30; 17 ♂♂, 13 ♀♀). See Suppl. material 1.

#### Diagnosis.

*Australosagolahelenae* comb. nov. can be distinguished from other *Australosagola* by the following characters: abdominal sternite 5 (VII) with small median, preapical tubercle (Fig. 5E, white arrow), lacking preapical setal rows (Fig. 5E, K).

#### Male description.

Length 2.2–2.7 mm. Body brown to reddish-brown. ***Head*.** Head in dorsal view with concavity at base of frontal rostrum around frontal fovea. Vertexal foveae well-developed (Fig. 5C, H). Antennomere 1 cylindrical and longer than wide; 2 subquadrate and longer than wide; 3 smallest and subconical; 4–7 subquadrate, longer than wide, swollen at middle; 8 subquadrate, as long as wide; 9 and 10 subquadrate and transverse (Fig. 5A, G). ***Thorax*.** Prothorax slightly broader than long, widest at midpoint (Fig. 5I). Elytra with two subbasal elytral foveae, three basal elytral foveae (1 being fovea at base of sutural stria), discal elytral foveae with short discal striae, and fovea in sutural striae. Hind wings fully developed. Metatrochanter with ventral margin convex (Fig. 5E, J). ***Abdomen*.** Abdominal tergite 2 (V) ~1/2 length of 3 (VI) (Fig. 1D). Abdominal sternite 5 (VII) slightly medially impressed (Fig. 5E). ***Genitalia*.** Length 0.32 mm, aedeagus symmetrical, apex of median lobe rounded and bulbous in ventral and basoventral views (Fig. 5L–N). Pair of edentate projections at midpoint of median lobe longer than parameres and not curved at apices in basoventral and lateral views, shortly curved in ventral view (Fig. 5L, M). Pair of tubercles dorsal to projection [Fig. 5L (black arrow), N (black arrow)]. Hook-shaped projection at basal part of median lobe only slightly curved in lateral view, projection appearing to be M-shaped and directed apically at basal part of median lobe in ventral view, phallobase narrowly rounded at apex in ventral view, shortly bent in lateral view (Fig. 5L–N).

#### Female sexual characters.

Abdominal sternites convex, apical portion of abdominal sternite 5 (VII) convex (Fig. 5F).

#### Comment.

*Australosagolahelenae* and *A.jiwooki* sp. nov. are the only species that have males with a small, median, preapical tubercle on abdominal sternite 5 (VII) [Figs 5E (white arrow), K (white arrow), 8H (white arrow)], while *A.helenae* is separated from *A.jiwooki* by the lack of the pair of setal clusters at the apex of the median projection at the posterior margin (Figs 5K, 8H, black arrow).

#### Distribution.

New South Wales, Victoria, and the Australian Capital Territory (Fig. 14, black hexagons).

#### Habitat.

Specimens of this species were collected using flight intercept traps (F.I.T.), spraying pyrethrin on logs, and by sifting leaf, log or forest floor litter in mallee, woodland, or most commonly in wet sclerophyll forests dominated by *Nothofaguscunninghami* and *Eucalyptus* species.

### ﻿ Australosagola minsangi sp. nov.

Taxon classificationAnimaliaColeopteraStaphylinidae

F3EBE37F-723E-5E11-B2E3-8F1696BDA530

https://zoobank.org/5F5CD962-2DAC-4221-B9A5-0761005ED82C

Figs 1E , 2D , 6 , 13

#### Type material.

***Holotype*. Australia: New South Wales**: • ♂ (ANIC), “AUSTRALIA:NWS, Styx / River St. For, Cedar / Pit Floral Res., 42 / km SE Wollomombi // IV-20/V-12-93, N-S / DSChandler, FIT old / temperate rainfor.”. ***Paratypes*** (*n* = 46; 29 ♂♂, 17 ♀♀). **Australia: New South Wales**: 12 ♂♂ 3 ♀♀ (2 ♂♂ slide-mounted; • 1 ♂ aedeagus dissected; FMNH), New England N.P., Robinson’s Knob Rd., 1 km E Pk. gate, *Noth.moorei* forest, slope, 1,320 m, 30°30'S, 152°24'E, 29 XII 1986–14 I 1987, FMHD #86-689, flight intercept (window) trap, A. Newton & M. Thayer 781; • 2 ♂♂ 1 ♀ (1 ♀ slide-mounted; FMNH), New England N.P., Robinson’s Knob Rd., 1 km E Pk. gate, *Noth.moorei* forest, 1,305 m, level, 30°30'S, 152°24'E, 29 XII 1986–14 I 1987, FMHD #86-686, flight intercept (window) trap, A. Newton & M. Thayer 780; • 1 ♀ (UNHC), Styx River S. F. N-S Brushwood Rd., 29 km SE Wollomombi, 960 m, 25 II–15 III 1993, FIT trap cut rainforest, D. S. Chandler; • 1 ♀ (slide-mounted; UNHC), Mt. Duval Fire Rd., 15 km NW Armidale, 1,320 m, 14 II 1993, sift leaf litter *Euc.laevopinea* dry sclerophyll, D. S. Chandler; • 1 ♀ (UNHC) Styx River S. F. Thru Road, 24 km SE Wollomombi, 980 m, 4 III 1993, sift *Eucalypt* & etc. leaf litter cut wet sclerophyll, D. S. Chandler; • 1 ♀ (UNHC), 15 km NW Armidale Mt. Duval, Tin Weir Crk., 1,200 m, 14–29 III 1993, FIT, dry sclerophyll, D. S. Chandler; • 1 ♀ (UNHC), Styx River St. For, Cedar Pit Floral Res., 40 km SE Wollomombi, 990 m, 6 XI–1 XII 1993, N–S FIT, old wet sclerophyll, K. MacGregor; • 1 ♀ (UNHC), Styx River St. For Cedar Pit Floral Res., 40 km SE Wollomombi, 990 m, 25 II–15 III 1993, N-S FIT trap old wet sclerophyll, D. S. Chandler; • 2 ♂♂ (UNHC), Styx River St. For, Cedar Pit Floral Res., 40 km SE Wollomombi, 990 m, 15 III 1993, *Eucal.*. & etc. leaf litter old wet sclerophyll, D. S. Chandler; • 1 ♂ 1 ♀ (UNHC), Styx River St. For, Cedar Pit Floral Res., 40 km SE Wollomombi, 990 m, 19 I–2 II 1994, N-S FIT, old wet sclerophyll, K. MacGregor; • 2 ♂♂ (UNHC), Styx River St. For, Cedar Pit Floral Res., 40 km SE Wollomombi, 990 m, 3–15 II 1994, E-W FIT, old wet sclerophyll, K. MacGregor; • 1 ♂ (aedeagus dissected; UNHC), Styx River St. For, Cedar Pit Floral Res., 40 km SE Wollomombi, 990 m, 22–24 II 1993, N-S FIT trap old wet sclerophyll, D. S. Chandler; • 1 ♂ (aedeagus dissected; UNHC), 40 km SW Singleton Darkey Crk., 17 III 1993, *Eucal.salligna* litter by stream, D. S. Chandler; • 1 ♂ (aedeagus dissected; UNHC), Styx River St. For, Cedar Pit Floral Res., 42 km SE Wollomombi, 935 m, 3–15 II 1994, N-S FIT, old rainforest, K. MacGregor; • 1 ♂ (aedeagus dissected; UNHC), Styx River St. For. Brushwood Rd., 29 km SE Wollomombi, 960 m, 3–15 II 1994, FIT trap E-W, cut rainforest, K. MacGregor; • 1 ♀ (UNHC), Styx River St. For, Cedar Pit Floral Res., 40 km SE Wollomombi, 990 m, 3–15 II 1994, N-S FIT, old wet sclerophyll, K. MacGregor; • 1 ♂ 2 ♀♀ (1 ♂ mounted in micro vial; UNHC), Mt. Wilson, 18 VII 1983, FMHD #83-276, rainforest litter nr. stream, L. E. Watrous; • 1 ♂ 1 ♀ (1 ♂ aedeagus dissected; UNHC), M. Wilson, 20 VII 1983, FMHD #83-279, rainforest litter nr. stream, L. E. Watrous; • 1 ♂ (slide-mounted; ANIC), Styx River St. For, Cedar Pit Floral Res., 40 km SE Wollomombi, 990 m, 25 II–15 III 1993, E-W FIT, old wet sclerophyll, D. S. Chandler; • 1 ♂ (ANIC), Styx River St. For, Cedar Pit Floral Res., 40 km SE Wollomombi, 990 m, 25 VI 1993, sift rotten old wet sclerophyll, D. S. Chandler; • 2 ♂♂ (ANIC), Styx River St. For, Thru Road, 24 km SE Wollomombi, 980 m, 3–15 II 1994, FIT trap E-W, cut wet sclerophyll, K. MacGregor; • 1 ♀ (ANIC), Styx River St. For, Cedar Pit Floral Res., 40 km SE Wollomombi, 990 m, 15 XII 1993–2 I 1994, N-S FIT, old wet sclerophyll, K. MacGregor; • 1 ♀ (ANIC), Styx River St. For, Cedar Pit Floral Res., 40 km SE Wollomombi, 990 m, 3–18 I 1994, N-S FIT, old wet sclerophyll, K. MacGregor.

#### Diagnosis.

*Australosagolaminsangi* sp. nov. can be easily distinguished from other species by the short and strongly transverse antennomeres (Fig. 6A, B, G), as well as antennomere 1 of both sexes having a pit on the dorsal surface (Fig. 6C, D, H, black arrows).

#### Male description.

Length 2.8–3.5 mm. ***Head*.** In dorsal view with concavity at base of frontal rostrum around frontal fovea. Rostrum broad and anterolaterally angulate (Fig. 6C). Antennomere 1 stout, subquadrate, and slightly longer than wide, slightly curved medially, with dorsal preapical fovea (Fig. 6A, G, H); 2 slightly longer than wide and subconical; 3 smallest, forming distinct disc and transverse; 4–10 subquadrate and transverse (Fig. 6A, G). ***Thorax*.** Prothorax slightly broader than long, widest at midpoint (Fig. 6I). Elytra with two subbasal elytral foveae, three basal elytral foveae (1 being fovea at base of sutural stria), discal elytral foveae with short discal striae, and fovea in sutural striae. Hind wings fully developed. Metatrochanter angulate on ventral margin (Fig. 6E, J). ***Abdomen*.** Abdominal sternites 4 and 5 (VI–VII) medially impressed (Fig. 6E), 5 (VII) with lateral preapical and apical rows of thick setae near middle (Fig. 6E, K). ***Genitalia*.** Length 0.46 mm, aedeagus symmetrical, pair of elongate projections at midpoint of median lobe widening to apices, longer than parameres, apices bluntly rounded (Fig. 6L–N). Apex of median lobe with small median spine visible in ventral and basoventral views (Fig. 6L, M, black arrows). In lateral view, projections at base of median lobe strongly curved (Fig. 6N), appearing broadly V-shaped in basolateral view and directed apically from basal margin of median lobe, lateral margins of median lobe spinose at middle in ventral view, phallobase margins bluntly rounded at middle in ventral view, in lateral view flat and shortly curved (Fig. 6L–N).

#### Female sexual characters.

Metatrochanter with posterior margin convex (Fig. 6F). Abdominal sternites 4–5 (VI–VII) convex, lacking thick setae at apex of abdominal sternite 5 (VII; Fig. 6F).

#### Etymology.

This species is named for a biological control specialist who has been an enthusiastic supporter of this study, Min-Sang Jang.

#### Distribution.

New South Wales (Fig. 13, black squares).

#### Habitat.

Specimens of this species were collected using flight intercept traps (F.I.T.), by sifting leaf or rotten woods in old and cut dry and wet sclerophyll *Eucalyptus* forests and in old warm-temperate rainforests.

### ﻿ Australosagola minhoi sp. nov.

Taxon classificationAnimaliaColeopteraStaphylinidae

3022EC4E-ACD4-5907-94A6-32578623CF15

https://zoobank.org/273BC9A5-CBBC-4944-8D0A-5B36331D66C2

Figs 1F , 2F , 7 , 14

#### Type material.

***Holotype*. Australia: New South Wales**: • ♂ (aedeagus dissected; ANIC), “AUSTRALIA:NSW., / Brown Mtn. Floral / Res., 0.5 km SSW / Cochrane Dam, 940 m // II-8/22-1993 / ANewton & MThayer / cool temp.rainfor / window trap”. ***Paratypes*** (*n* = 46; 37 ♂♂, 9 ♀♀). **Australia: New South Wales**: • 31 ♂♂ 6 ♀♀ (10 ♂♂ aedeagus dissected; 2 ♂♂ 1 ♀ slide-mounted; FMNH), Mt. Brown, Flora Res., 0.5 km SSW Cochrane Dam., 950 m, 36°35'S, 149°27'E, 20 XII 1986–15 II 1987, warm-temp. rainforest, FMHD #86-648, flight intercept (window) trap, A. Newton & M. Thayer 767; • 4 ♂♂ 2 ♀♀ [4 ♂♂ 1 ♀ (1 ♂ aedeagus dissected; 1 ♂ slide-mounted; FMNH), 1 ♀ (UNHC)], same data as holotype; • 1 ♀ (slide-mounted; FMNH), Brown Mt., Flora Res., 0.5 km SSW Cochrane Dam, warm-temp. rainforest, 950 m, 36°35'S, 149°27'E, 20 XII 1986, FMHD #86-650, berl., leaf & log litter, forest floor, A. Newton & M. Thayer 767; **Tasmania**: • 1 ♂ (ANIC), Claytons, Bathurst Harbour, 43°22'S, 146°08'E, 7 XII 1990–15 I 1991, F.I.T. #3 F.I.T. ANIC 1149 closed forest litter #3, E. Nielsen, T. Edwards; • 1 ♂ (ANIC, slide-mounted), Claytons, Bathurst Harbour, 43°22'S, 146°08'E, 29 VIII–28 XI 1991, F.I.T. #3, F.I.T. ANIC 1204 closed forest, I. Naumman, G. Clarke.

#### Diagnosis.

Male specimens of *A.minhoi* sp. nov. can be distinguished from the other *Australosagola* species by the large and prominent eyes (Fig. 7C, H) and the broad apex of the median lobe of the aedeagus best seen in a ventral and basoventral views (Fig. 7L, M, black arrow).

#### Male description.

Length 2.7–3.0 mm. Body reddish to dark brown. ***Head*.** Head in dorsal view with concavity at base of frontal rostrum around frontal fovea; frontal sulcus slightly separated from base to apex (Fig. 7H, black arrow). Vertexal foveae well-developed (Fig. 7C, H). Antennomere 1 cylindrical and longer than wide; 2 subquadrate and longer than wide; 3 smallest, subconical and slightly longer than wide; 4–6 subquadrate, slightly longer than wide; 7 and 8 trapezoidal, as long as wide; 9 and 10 subquadrate and transverse (Fig. 7A, G). ***Thorax*.** Prothorax slightly broader than long (Fig. 7I). Elytra with two subbasal elytral foveae, three basal elytral foveae (1 being fovea at base of sutural stria), discal elytral foveae with short discal striae, and fovea in sutural striae. Hind wings fully developed. Metatrochanter with ventral margin angulate (Fig. 7E, J). ***Abdomen*.** Abdominal sternites 4 and 5 (VI–VII) broadly and medially impressed (Fig. 7E). Abdominal sternite 5 (VII) with lateral rows of apical setae near middle, apex of sternite VII indented at middle (Fig. 7K). ***Genitalia*.** Length 0.45 mm, aedeagus symmetrical, apical portion of median lobe abruptly widened to appear as spines in ventral view, then narrowed to broad apex, this area broadly rounded and with separated apices in basoventral view (Fig. 7M, black arrow). Single tiny tubercle at apex of median lobe in basoventral view (Fig. 7M, white arrow). Pair of dentate projections at midpoint of median lobe curved slightly medially at apices in basoventral view, longer than parameres (Fig. 7L, M). Projections at basal part of median lobe strongly curved in lateral view, appearing broadly V-shaped in basolateral view and directed apically, phallobase with lateral margins unevenly rounded in ventral view, shortly curved in lateral view (Fig. 7L–N).

#### Female sexual characters.

Antennomeres 9 and 10 wider than those of male (Fig. 7B). Metatrochanter with posterior margin convex (Fig. 7F). Abdominal sternites convex, lacking apical rows of setae on abdominal sternite 5 (VII; Fig. 7F).

#### Comment.

Aedeagi of *A.rugicornis*, *A.tasmaniae*, *A.minsangi*, *A.minhoi*, and *A.jungjooni* are similar in general appearance, with *A.minhoi* having the widest median lobe (Fig. 7M, black arrow). This species can be easily separated from any other species in *Australosagola* by the large, prominent eyes (Fig. 7H). Additionally, *A.tasmaniae* and *A.rugicornis* each possess two rows of setal clusters on abdominal sternite 5 (VII), whereas *A.minhoi* has only a single row (Figs 3N, 4K, 7K).

#### Etymology.

This species is named for a specialist in aquatic insect ecology who has been an enthusiastic supporter of this study, Min-Ho Song.

#### Distribution.

New South Wales and Tasmania (Fig. 14, blue triangles).

#### Habitat.

Specimens of this species were collected using flight intercept traps (F.I.T.), and by sifting leaf, log, or forest floor litter. Found most commonly in warm-temperate rainforests.

### ﻿ Australosagola jiwooki sp. nov.

Taxon classificationAnimaliaColeopteraStaphylinidae

E31E2FD6-1083-5B04-9AE5-E85324CF230B

https://zoobank.org/1DDF2D6C-C721-45FD-B2D3-7ADBABA6EF8E

Figs 1G , 8 , 14

#### Type material.

***Holotype*. Australia: Victoria**: • ♂ (aedeagus dissected; ANIC), “AUSTL.: VIC.: Mt. Buffalo / N.P., above Eurobin Point / 820 m 36.42S 146. 50E / 23.I.1987 / wet sclerophyll forest // A.Newton & M.Thayer 805 / FMHD #87-202 / berl., leaf & log / litter, forest floor” (actual coordinate data: 36°42'S, 146°50'E = -36.70, 146.83). ***Paratype*** (*n* = 1; 1 ♂). **Australia: Victoria**: • 1 ♂ (slide-mounted; FMNH), same data as holotype.

#### Diagnosis.

*Australosagolajiwooki* sp. nov. can be distinguished from other *Australosagola* species by the weakly defined frontal fovea (Fig. 8B, E, white arrows), and by the combination of a median preapical tubercle and small mediolateral clusters of spines at the apex of male sternite 5 (VII; Fig. 8C, H).

#### Male description.

Length 2.4 mm. Body reddish-brown. ***Head*.** Head in dorsal view, shallowly impressed at base of frontal rostrum (Fig. 8B, E). Vertexal foveae well-developed (Fig. 8B, E). Antennomere 1 subconical and longer than wide; 2 rounded and longer than wide; 3 smallest, subquadrate and transverse; 4–6 subquadrate and longer than wide; 7 subquadrate and as long as wide; 8 subquadrate and transverse; 9 subquadrate and as long as wide; 10 trapezoidal and transverse (Fig. 8A, D). ***Thorax*.** Prothorax slightly broader than long (Fig. 8F). Elytra with two subbasal elytral foveae, three basal elytral foveae (1 being fovea at base of sutural stria), discal elytral foveae with short discal striae, and fovea in sutural striae. Hind wings fully developed. Metatrochanter with ventral margin convex (Fig. 8C, G). ***Abdomen*.** Only abdominal sternite 5 (VII) with median impression (Fig. 8C), 5 (VII) with pair of setal clusters at apex of median projection of posterior margin (Fig. 8C, H, black arrows), with median, preapical tubercle (Fig. 8C, H, white arrows). ***Genitalia*.** Length 0.48 mm, aedeagus symmetrical, in basovental view with lateral margins roughly parallel, in lateral view apical 1/2 of median lobe greatly inflated; median lobe with lateral margins sub-angulate at apex in basoventral view (Fig. 8J), pair of projections at midpoint of median lobe straight in lateral view, slightly sinuate in ventral view, slightly longer than parameres (Fig. 8J, K). Tubercles at base of median lobe bent near base in lateral view, thickly V-shaped in ventral view (Fig. 8I, K). Phallobase small with lateral margins evenly rounded in ventral view, flat, short, and evenly curved in lateral view (Fig. 8I, K).

#### Female sexual characters.

Female unknown.

#### Comment.

*Australosagolajiwooki* has a small frontal fovea that is slightly impressed at the base of the frontal rostrum around the frontal fovea (Fig. 8B). This species also has a unique modified form at the apex of abdominal sternite 5 (VII), a pair of median apical setal clusters that have only 6–8 setae together with a preapical median tubercle (Fig. 8H, white and black arrows).

#### Etymology.

This species is named for a curculionid specialist who has been an enthusiastic supporter of this study, Ji-Wook Kim.

#### Distribution.

Victoria (Fig. 14, red square).

#### Habitat.

Both specimens were collected by sifting leaf, log, and forest floor materials in a wet sclerophyll forest.

### ﻿ Australosagola sunheei sp. nov.

Taxon classificationAnimaliaColeopteraStaphylinidae

62B4359D-43A7-5407-85F1-10D658600847

https://zoobank.org/9DE01301-F174-4E98-9BBA-8B19030BD7ED

Figs 1H , 2E , 9 , 13

#### Type material.

***Holotype*. Australia: South Australia**: • ♂ (aedeagus dissected; ANIC), “AUSTL:S.Austl., 10 / km SE Adelaide, / Belaire Rec. Pk., / 29-VI-1983 // FMHD #83-248, damp / leaf litter, L.E. / Watrous”. ***Paratype*** (*n* = 1; 1 ♀). **Australia: South Australia**: • 1 ♀ (slide-mounted; UNHC), same data as holotype.

#### Diagnosis.

*Australosagolasunheei* sp. nov. can be distinguished by the following characters: head being widest at tempora (Fig. 9C, D, H), median lobe of aedeagus with prominent broad projection at middle (Fig. 9N, black arrow).

#### Male description.

Length. 2.3–2.5 mm. ***Head*.** Head with frontal sulcus and area around frontal fovea concave in dorsal view. Vertexal foveae well-developed (Fig. 9C). Antennomere 1 cylindrical and longer than wide; 2 slightly subconical and longer than wide; 3 subconical, smallest and slightly longer than wide; 4–8 subquadrate and as long as wide; 9 and 10 subquadrate and transverse (Fig. 9A, G). ***Thorax*.** Prothorax slightly broader than long, widest at midpoint (Fig. 9I). Elytra with two subbasal elytral foveae, three basal elytral foveae (one being fovea at base of sutural stria), discal elytral foveae with short discal striae, and fovea in sutural striae. Metatrochanter with posterior margin smoothly convex (Fig. 9E). ***Abdomen*.** Only abdominal sternite 5 (VII) medially impressed; with pair of setal clusters at apex of median projection (Fig. 9E). Abdomen with visible tergite 2 (V) ~2/3 length of 3 (VI) (Fig. 1H). ***Genitalia*.** Length 0.36 mm, aedeagus symmetrical, projection of median lobe broadly expanded laterally at middle in basoventral view (Fig. 9N, black arrow), projection longer than parameres (Fig. 9O). Projections at base slightly curved in lateral view, heart-shaped (chordate) in ventral view, phallobase with lateral margins evenly rounded in ventral view, flat, short, and evenly curved in lateral view (Fig. 9M–O).

#### Female sexual characters.

Eyes smaller than those of male (Fig. 9D). Hind wings comparatively small (Fig. 9J). Abdominal sternites convex (Fig. 9F); lacking setae at apex of abdominal sternite 5 (VII; Fig. 9F, L).

#### Comment.

*Australosagolasunheei* can be separated from allied *A.yongsooni* by the features of the aedeagus, which in *A.sunheei* has a broader median projection of the median lobe than does *A.yongsooni* (Figs 9N (black arrow), 12N).

#### Etymology.

This species is named for a respected mentor of the first author who is a specialist in biological control, Dr. Sun-Hee Hong.

#### Distribution.

South Australia (Fig. 13, black hexagon).

#### Habitat.

Specimens of this species were collected from damp leaf litter.

### ﻿ Australosagola doohyungi sp. nov.

Taxon classificationAnimaliaColeopteraStaphylinidae

7EEBB0EC-CE6E-5F8C-B418-71F4E25C500A

https://zoobank.org/6B23A546-BC19-41A0-AC99-9677AD7046E4

Figs 1I , 2G , 10 , 14

#### Type material.

***Holotype*. Australia: Western Australia**: • ♂ (ANIC), “AUSTRALIA:WAust., / Nornalup, Valley / of Giants, VI-20-80 / S&JPeck, berl. / tingle tree bark”. ***Paratypes*** (*n* = 37; 19 ♂♂, 18 ♀♀). **Australia: Western Australia**: • 3 ♂♂ 1 ♀ (1 ♂ aedeagus dissected; 1 ♂ slide-mounted; UNHC), same data as holotype; • 5 ♂♂ 2 ♀♀ (1 ♂ aedeagus dissected; 1 ♂ slide-mounted; UNHC), Windy Harbour, 27 km S Northcliffe, 8 VII 1980, coastal scrub litter, S. & J. Peck; • 3 ♂♂ 2 ♀♀ (1 ♂ aedeagus dissected; 1 ♂ slide-mounted; UNHC), 40 km ESE Manjimup, 15 VII 1980, jarrah forest litter, S. & J. Peck; • 1 ♂ 3 ♀♀ (UNHC), Pemberton, Warren N.P., 5 VII 1980, berl. Karri litter & fungi, S. & J. Peck; • 1 ♂ 2 ♀♀ (1 ♂ aedeagus dissected; UNHC), Walpole N.P., “Tingle Tree”, 18–27 VI 1980, berl. Fungi & litter, S. & J. Peck; • 1 ♀ (UNHC), 50 km SW Nannup Sues Bridge, 26 VII 1980, berl. Marri log & leaf litter, S. & J. Peck; • 1 ♀ (UNHC), Walpole N.P., “Tingle Tree”, 18–27 VI 1980, FIT in dense bush, S. & J. Peck; • 1 ♂ 3 ♀♀ (1 ♂ aedeagus dissected; FMNH), Warren N.P., Maidenbush Tr., old-growth karri forest (*Euc.diversicolor*), 60 m, 34°30.515'S, 115°57.411'E, 29 VII 2004, FMHD #2004-113, berl., leaf & bark litter, Newton, Clarke 1104; • 1 ♀ (FMNH), Warren N.P., Maidenbush Tr., old-growth karri forest (*Euc.diversicolor*), 60 m, 34°29.73'S, 115°58.62'E, 29 VII 2004, FMHD #2004-154, berl., leaf & bark litter, D. Clarke, M. Thayer, A. Newton 1105; • 1 ♂ (FMNH), aedeagus dissected and mounted in euparal on a clear plastic card, Beedelup N.P., Walk-though Tree vic., karri forest (*Euc.diversicolor*), 100 m, 34°25.7'S, 115°58.63'E, 30 VII–10 VIII 2004, FMHD #2004-116, flight intercept trap, A. Newton, M. Thayer, A. Solodovnikov 1106; • 1 ♂ 1 ♀ (1 ♀ slide-mounted; FMNH), Shannon N.P., Big Trees Grove vic., karri forest (*Eucalyptusdiversicolor*), 140 m, 34°37.84'S, 116°19.81'E, 30 VII–11 VIII 2004, FMHD #2004-122, flight intercept trap, A. Newton, A. Solodovnikov, M. Thayer 1108; • 1 ♀ (slide-mounted; FMNH), Beedelup N.P., Beedelup Falls Rd., jarrah (*Eucalyptusmarginata*) forest with *Banksiagrandis*, *Xanthorrhoea*, 150 m, 34°25.81'S, 115°53.098'E, 31 VII–11 VIII 2004, FMHD #2004-126, flight intercept trap, Newton, Solodovnikov, Thayer 1109; • 1 ♂ (aedeagus dissected; FMNH), Walpole-Nornalup N.P., Giant Tingle Tree area, tingle-*Allocasuarina*-karri (*Eucalyptusdiversicolor*) forest, 190 m, 34°58.88'S, 116°47.42'E, 2–9 VIII 2004, FMHD #2004-130, flight intercept trap, Newton, Solodovnikov, Thayer 1110; • 1 ♂ (aedeagus dissected; FMNH), Walpole N.P., Z19—Z09 Rd., 20 VI–4 VII 1980, FMHD #80-391 bracket fungi & litter, S. & J. Peck; • 1 ♂ (aedeagus dissected; ANIC), Walpole N.P., “Tingle Tree” 18–27 VI 1980, malaise traps with trough dense bush, S. & J. Peck SBP59.

#### Diagnosis.

*Australosagoladoohyungi* sp. nov. can be distinguished from other *Australosagola* species by the median lobe of the aedeagus being constricted at the midpoint (Fig. 10L), and with this being the only species lacking the projections at the midpoint of the median lobe (Fig. 10L–N).

#### Male description.

Length. 2.0–2.3 mm. ***Head*.** Head slightly broader than long; frontal sulcus with contiguous margins, area posterior to frontal rostrum impressed around frontal fovea (Fig. 10C, H). Vertexal foveae well-developed (Fig. 10C, H). Tempora posterolaterally angulate. Antennomere 1 longer than wide; 2 subquadrate and longer than wide; 3 subconical and longer than wide; 4–7 subquadrate and longer than wide; 8 somewhat globose and longer than wide; 9 and 10 somewhat globose and slightly longer than wide (Fig. 10A, G). ***Thorax*.** Prothorax slightly broader than long, widest at midpoint (Fig. 10I). Elytra with two subbasal elytral foveae, three basal elytral foveae (1 being fovea at base of sutural stria), discal elytral foveae with short discal striae, and fovea in sutural striae. Hind wings fully developed. Metatrochanter with ventral margin slightly angulate (Fig. 10E, J). ***Abdomen*.** Abdominal sternites 4 and 5 (VI–VII) broadly convex (Fig. 10E, K). Tergite 2 (V) ~1/2 length of 3 (VI) (Fig. 10E, K). Abdominal sternite 5 (VII) lacking modifications. ***Genitalia*.** Length 0.39 mm, aedeagus symmetrical, with basal 1/2 of median lobe slender, constricted to middle, apical 1/2 expanded, widest at apical 1/3, then narrowing to subtruncate apex with blunt median point, in lateral view apex elongate and acutely pointed (Fig. 10N), with pair of blunt preapical spines (Fig. 10L, black arrow). Preapex of median lobe with apically divided small and blunt process best seen in basoventral and lateral views (Fig. 10M, N, black arrows). Parameres long and extending almost to apex of median lobe, projection at base of median lobe evenly curved apically, phallobase in ventral view with margins evenly rounded (Fig. 10L, M), in lateral view flattened, slightly curved (Fig. 10M).

#### Female sexual characters.

Head as long as wide (Fig. 10D). Metatrochanter with posterior margin convex (Fig. 10F).

#### Comment.

This species has a unique aedeagus that lacks projections at the midpoint of the median lobe. It could be that the median lobe is only expanded at the apex of the median lobe, so that the small and blunt end projection at the apex of the median lobe could be the ancestral or derived version of this projection, which is seen in other species.

#### Etymology.

This species is named for respected mentor of the first author who is a specialist in ecology and ethology, Dr. Doo-Hyung Lee.

#### Distribution.

Western Australia (Fig. 14, blue circles).

#### Habitat.

Specimens of this species were collected by malaise traps, flight intercept traps (F.I.T.), or by sifting bark, leaf, fungi, or logs in wet sclerophyll *Eucalyptus* forests, or by sifting coastal scrub litter.

### ﻿ Australosagola jungjooni sp. nov.

Taxon classificationAnimaliaColeopteraStaphylinidae

A5B8BB60-BBA8-5901-A73C-51D03DCC1A17

https://zoobank.org/BD7AB3B5-8702-4E6D-846C-B9AFCFF7222C

Figs 1J , 2H , 11 , 13

#### Type material.

***Holotype*. Australia: New South Wales**: • ♂ (aedeagus dissected; ANIC), “AUSTALIA: NSW, / Kosciusko Nat. Park / 13 km NW Jindabyne / 1 km W Sawpit Crk. // Cmpgd., 1240 m, V-2- / 1993, DSChandler / sift basal litter / Euc. dalrympleana”. ***Paratypes*** (*n* = 2; 2 ♀♀). **Australia: New South Wales**: • 1 ♀ (slide-mounted; UNHC), Kosciusko Nat. Park 13 km NW Jindabyne 1 km W Sawpit Crk. Cmpgd., 1,240 m, 2 V 1993, *Euc.dalrympleana*, *Euc.pauciflora*, & grass litters, D. S. Chandler; • 1 ♀ (FMNH), Mt. Brown, Flora Res., 0.5 km SSW Cochrane Dam, warm-temp, rainforest, 950 m, 36°35'S, 149°27'E, 20 XII 1986–15 II 1987, FMHD #86-648, flight intercept (window) trap, A. Newton & M. Thayer 767.

#### Diagnosis.

This species can be distinguished from other species by the following characters: antennomere 3 transverse, 4–7 subquadrate (Fig. 11A, B, G), apex of aedeagus lacking sharp projection in lateral view (Fig. 11N, black arrow).

#### Male description.

Length. 2.2–2.3 mm. ***Head*.** Head with margins of frontal sulcus contiguous, area posterior to frontal rostrum deeply impressed around frontal fovea; broader than long, widest across eyes (Fig. 11C). Vertexal foveae well-developed (Fig. 11C). Antennomere 1 cylindrical and longer than wide; 2 subquadrate and longer than wide; 3 smallest, subquadrate and flattened; 4–8 subquadrate and as long as wide; 9 and 10 subquadrate and transverse (Fig. 11A). ***Thorax*.** Prothorax as long as wide. Elytra with three basal elytral foveae (1 being fovea at base of sutural stria), discal elytral foveae with short discal striae, and fovea in sutural striae. Metatrochanter with ventral margin angulate (Fig. 11E). ***Abdomen*.** Abdominal sternites 4 and 5 (VI–VII) largely medially impressed (Fig. 11E). Abdominal tergite 2 (V) ~2/3 length of tergite 3 (VI; Figs 1J, 2H). ***Genitalia*.** Length 0.44 mm, aedeagus symmetrical, preapex of median lobe laterally angulate, then evenly convergent to broad truncate apex with median point in ventral view (Fig. 11L, black arrow), in basoventral view apical 1/3 broadly lobed with apices broadly and bluntly rounded (Fig. 11M). Apex of median lobe with small median spine visible in ventral view (Fig. 11L, black arrow); area inflated and broadly rounded in lateral view (Fig. 11N); W-shaped projection at base of median lobe distinct in ventral and basoventral views (Fig. 11L, M); phallobase with lateral margins broadly rounded (Fig. 11L, M), slightly curved in lateral view, with basal margin hooked (Fig. 11N).

#### Female sexual characters.

Antennomere 1 cylindrical and longer than wide; 2 slightly subconical and longer than wide; 3 smallest, transverse; 4–10 larger and transverse (Fig. 11B, G).

#### Comment.

Because only a single male specimen was available, we could not confirm the presence of subbasal elytral foveae. However, in females we were able to confirm the presence of two subbasal elytral foveae, as is found in the other species. Specimens of *A.jungjooni* sp. nov. can be distinguished from other *Australosagola* species, except *A.minsangi*, by the presence of transverse antennomere 3 for both sexes. *Australosagolajungjooni* sp. nov. can be separated from *A.minsangi* by antennomeres 4–7 of *A.jungjooni* being more subquadrate, while these antennomeres are greatly transverse, almost disc-like, for *A.minsangi* (Figs 6A, B, G, 11A, B, G). Also, the aedeagus of *A.jungjooni* resembles that of *A.minsangi*, but the apical part of the aedeagus in lateral view is more acutely projecting in *A.minsangi* (Fig. 6N), while for *A.jungjooni* it is quite bulbous (Fig. 11N, black arrow).

#### Etymology.

This species is named for a respected mentor of the first author, an insect ecology specialist, Dr. Jung-Joon Park.

#### Distribution.

New South Wales (Fig. 13, black stars).

#### Habitat.

Specimens of this species were collected by sifting leaf and grass litters at the base of *Eucalyptus* trees, or were taken by flight intercept traps (F.I.T.) in *Eucalyptus* woodlands or warm-temperate rainforests.

### ﻿ Australosagola yongsooni sp. nov.

Taxon classificationAnimaliaColeopteraStaphylinidae

2DA97A78-5F7F-51C7-A486-698873801667

https://zoobank.org/7D97219F-94D3-4E66-9F3C-E2C68265521D

Figs 1K , 2I , 12 , 14

#### Type material.

***Holotype*. Australia: South Australia**: • ♂ (aedeagus dissected; ANIC), “AUSTRALIA:S.Aust., / 16 km SE Adelaide, / Cleland Conservat. / Pk.,Pill Box Track // 500 m, IV-25-1993 / DSChandler, grass / & Eucalypt litter / cut dry sclerophyl” ***Paratypes*** (*n* = 6; 2 ♂♂, 4 ♀♀). **Australia: South Australia**: • 3 ♀♀ (1 ♀ slide-mounted; UNHC), same data as holotype; • 1 ♂ 1 ♀ (1 ♂ slide-mounted; SAMA), Mt. Lofty summit, 26 VI 1988, soil & litter under *Euc.obiqua*, R. V. Southcott TX284; 1 ♂ (aedeagus dissected; SAMA), Mt. Lofty Rgs., in moss, R. J. Burton.

#### Diagnosis.

*Australosagolayongsooni* sp. nov. can be distinguished from other *Australosagola* species by its short elytra. Although the overall body size of *A.yongsooni* is comparable to that of other congeners, its elytra are distinctly shorter, measuring only 0.44 mm in length, whereas those of other species typically range from 0.57–0.88 mm (Figs 2I, 12J).

#### Male description.

Length. 1.8–2.2 mm. ***Head*.** Head broader than long, widest across eyes. Head with frontal sulcus and frontal fovea continuous, frontal sulcus widening posteriorly to form teardrop shape (Fig. 12C, white arrow, H). Vertexal foveae well visible ventrally, but indistinct dorsally (Fig. 12C, H). Antennomere 1 longer than wide; 2 subquadrate and longer than wide; 3 subconical, smallest, and as long as wide; 4–8 subquadrate and as long as wide; 9 and 10 subquadrate, wider than long (Fig. 12A, G). ***Thorax*.** Prothorax as long as wide (Fig. 12I). Elytra short 0.44 mm, as long as wide (Fig. 12J); two subbasal elytral foveae, three basal elytral foveae (1 being fovea at base of sutural stria), discal elytral foveae with short discal striae, and fovea in sutural striae (Fig. 12J). Hind wings fully developed (Fig. 12L). Metatrochanter with ventral margin convex (Fig. 12E, K). Lateral metaventral foveae enlarged (Fig. 12K, black arrow). ***Abdomen*.** Only abdominal sternite 5 (VII) medially concave, with pair of short setal rows at apex of median projection (Fig. 12E, M). Abdominal tergite 2 (V) ~2/3 length of tergite 3 (VI). ***Genitalia*.** Length 0.37 mm, aedeagus symmetrical, relatively narrow, apical 1/2 comparatively weakly sclerotized (Fig. 12N, O). Projections from midpoint straight, apices slightly divergent in ventral and basoventral views (Fig. 12N, O), longer than parameres (Fig. 12P). V-shaped projection at base of median lobe fused at base in ventral view, straight in lateral view, phallobase short, indistinct, with lateral margins evenly curved in ventral view (Fig. 12N–P).

#### Female sexual characters.

Antennomere 1 thicker than that of male and longer than wide; 2 subquadrate and longer than wide; 3 subconical, smallest, and as long as wide; 4–6 subquadrate and as long as wide; 7–10 subquadrate and transverse (Fig. 12B). Abdominal sternite 5 (VII) lacking median impression and setal rows (Fig. 12F).

#### Comment.

Although this species has short elytra, the male has long hind wings, and the relatively narrow aedeagus with the straight basal and medial projections of the median lobe that are both forked apically allow the species to be readily identified.

#### Etymology.

This species is named a respected mentor of the first author, a plant-microorganism interaction specialist, Dr. Yong-Soon Park.

#### Distribution.

South Australia (Fig. 14, black star).

#### Habitat.

Specimens of this species were collected by sifting soil, grass, and *Eucalyptus* litter beneath *Eucalyptus* trees in dry sclerophyll forests.

## Supplementary Material

XML Treatment for Australosagola

XML Treatment for Australosagola tasmaniae

XML Treatment for Australosagola rugicornis

XML Treatment for Australosagola helenae

XML Treatment for Australosagola minsangi

XML Treatment for Australosagola minhoi

XML Treatment for Australosagola jiwooki

XML Treatment for Australosagola sunheei

XML Treatment for Australosagola doohyungi

XML Treatment for Australosagola jungjooni

XML Treatment for Australosagola yongsooni

## ﻿Additional information

### Use of AI

No use of AI was reported.

### Author ORCIDs

Su-Ho Choi https://orcid.org/0009-0009-5105-5582

Jong-Seok Park https://orcid.org/0000-0003-2063-2534

### Data availability

Additional label data for *Australosagolatasmaniae*, *A.rugicornis*, and *A.helenae* can be found in Suppl. material 1.

## Supplementary materials

Supplementary material 1 Additional label data for *Australosagolatasmaniae*, *A.rugicornis*, and *A.helenae* Data type pdf https://binary.pensoft.net/file/1374255This dataset is made available under the Open Database License (http://opendatacommons.org/licenses/odbl/1.0/). The Open Database License (ODbL) is a license agreement intended to allow users to freely share, modify, and use this Dataset while maintaining this same freedom for others, provided that the original source and author(s) are credited.Su-Ho Choi, Donald S. Chandler, Jong-Seok Park
