# Supplementary material for: ﻿Australosagola, a new genus of pselaphine beetles from southern Australia (Coleoptera, Staphylinidae, Pselaphinae, Faronitae) with descriptions of seven new species
Source: Zookeys. 2025 Jul 15;1245:177–205. doi: 10.3897/zookeys.1245.151556 (PMC12284585; doi:10.3897/zookeys.1245.151556)
Supplement: Supplementary material 1 — Additional label data for Australosagolatasmaniae, A.rugicornis, and A.helenae [file zookeys-1245-177_article-151556__-s001.pdf]

Australosagola tasmaniae **(Lea, 1911), comb. nov.**

Other material examined (*n* = 164; 110 ♂♂, 54 ♀♀). **Australia: Australian Capital Territory:** 2♂♂ (ANIC), Blundells Creek, 3 km E of Piccadilly Circus, 850 m, 35°22'S 148°50'E, I 1984, flight intercept window/trough trap, Weir, Lawrence, Johnson; 2♂♂ (1♂ aedeagus dissected; ANIC), Blundells Creek, 3 km E of Piccadilly Circus, 850 m, 35°22'S 148°50'E, II 1984, flight intercept window/trough trap, Weir, Lawrence, Johnson; 1♀ (ANIC), Blundells Creek, 3 km E of Piccadilly Circus, 850 m, 30 III 1984, Berlesate ANIC 938, closed forest litter, Lawrence, Stevens, Johnson; 1♂ (aedeagus dissected; ANIC), Wombat Creek, 6 km NE of Piccadilly Circus, 750 m, 35°19'S 148°51'E, VI 1984, Berlesate ANIC 999, open forest, Weir, Lawrence, Johnson; 1♂ (ANIC), Wombat Creek, 6 km NE of Piccadilly Circus, 750 m, 35°19'S 148°51'E, I 1985, flight intercept window/trough trap, Weir, Lawrence, Johnson; 1♂ (ANIC), Blundells Creek, 3 km E of Piccadilly Circus, 850 m, 35°22'S 148°50'E, II 1985, flight intercept window/trough trap, Weir, Lawrence, Johnson; 3♂ (ANIC), Blundells Creek, 3 km E of Piccadilly Circus, 850 m, 35°22'S 148°50'E, III 1985, flight intercept window/trough trap, Weir, Lawrence, Johnson; 2♀♀ (ANIC), Blundells Creek, 3 km E of Piccadilly Circus, 850 m, 35°22'S 148°50'E, V 1985, flight intercept window/trough trap, Weir, Lawrence, Johnson; 1♀ (ANIC), Honeysuckle Creek, 35°35'S 149°00'E, 21–31 III 1985, Malaise trap/ethanol, I. Naumann, J. Cardale; 1♀ (ANIC), Blundells Creek, 3 km E of Piccadilly Circus, 850 m, 35°22'S 148°50'E, VI 1985, flight intercept window/trough trap, Weir, Lawrence, Johnson; 1♂ (UNHC), Mt. Ginini, 1,660 m, 35°32'S 148°46'E, leaf litter & moss, ANIC Berl. #659, J. Lawrence & T. Weir; 1♂ 5♀♀ (1♂ aedeagus dissected; ANIC), Blundells Ck., 3 km, E of Piccadilly Circus rainforest, 850 m, 35°22'S 148°50'E, 7 IX 1986, Berlesate ANIC 1066 leaf litter, J. F. Lawerence; 1♀ (UNHC), Mt. Brindabella, Blundell Creek Rd., 18 XI 1988, berl. leaf litter, J. & R. Bell; 1♂ (UNHC), Mt. Brindabella, Blundell Creek Rd., 2 XI 1988, pit trap, J. & R. Bell. **New South Wales:** 19♂♂ 3♀♀ (3♂♂ aedeagus dissected; 1♀ slide-mounted; FMNH), Kosciusko N.P., Leather Barrel Ck. picnic Area (0.6 km SW). 1,060 m, 36°32'S 148°11'E 7–21 II 1993, open *Eucalyptus* forest (gum + *delegatensis*) with shrubby understory, FMHD #93-68 window trap, A. Newton & M. Thayer 920; 1♂ (aedeagus dissected; FMNH), Currowan S.F. (NW Nelligen), Wallaby Forest Pres., 400 m, 35°33'S 150°02'E, 18 II 1987, wet sclerophyll

forest, berl., leaf & log litter, forest floor A. Newton & M. Thayer 771, FMHD #87-152; 2♂♂ (1♂ aedeagus dissected; FMNH), Macquarie Pass N.P., NW corner, 490 m, 34°33'S 150°39'E, 23 XII 1986, subtropical rainforest, berl., leaf & log litter, forest floor, A. Newton & M. Thayer 772, FMHD #86-662; 2♂♂ 1♀ (1♂ aedeagus dissected; FMNH), Macquarie Pass N.P., Clover Hill Rd., 290 m; 34°34'S 150°39'E, 23 XII 1986–18 I 1987 subtropical rainforest, flight intercept (window) trap, A. Newton & M. Thayer 773, FMHD #86-663; 1♀ (FMNH), Mt. Brown, nr. Nimnitabel, 620 m, Rutherford Creek, 26 V 1970, rainforest ANIC Berl., #285-6, RWT & R. Bartell; 2♀♀ (FMNH), Kosciusko N.P., 4.1 km W Dead Horse Gap, 1,500 m; 36°19'S 148°18'E, 19 XII 1986*, Euc. pauciflora* woodland, FMHD #86-647 berl., leaf & log litter, forest floor, A. Newton & M. Thayer 766; 1♂ (aedeagus dissected; FMNH), Kosciusko N.P., 4.1 km W Dead Horse Gap, 1,500 m, 36°32'S 148°13'E, 19 XII 1986–14 II 1987, *Euc. pauciflora* woodland, FMHD #86-645, flight intercept (window) trap, A. Newton & M. Thayer 766; 5♂♂ 1♀ [3♂♂ 1♀ (1♂ 1♀ slide-mounted; UNHC), 2♂♂ (1♂ aedeagus dissected; 1♂ slide-mounted; FMNH)], Mt. Clyde, 18 km SE Braidwood, 500 m, 28 V 1978, log litter & fungi wet sclerophyll, ravines, S & J. Peck; 1♂ (aedeagus dissected; FMNH), Monga State Forest, 700 m, 19–24 I 1984, *Eucalyptus* forest, on ferns, L. Masner; 1♂ 4♀♀ (UNHC), Mt. Brindabella, Lee's Spring, 21 XII 1986, Burckhardt; 1♀ (UNHC), Mt. Brindabella, Piccadilly Circus, 21 XII 1986, Burckhardt. **Tasmania:** 1♀ (slide-mounted; ANIC), Melaleuca near Bathurst Harbour, 43°25'S 146°10'E, 3–7 XII 1990, Winkler ANIC 1139 closed forest litter, T. Weir, I. Naumann; 1♀ (ANIC), Big Sassy Creek, 42°42'S 147°52'E, 12 V 1989, Tube 137, pyrethrin knockdown *Atherospermum* J. Diggle; 1♂ (aedeagus dissected; ANIC), Big Sassy Creek, 42°42'S 147°52'E, 12 V 1989, Tube 139, leaf litter rainfor. P. Greenslade; 1♂ (aedeagus dissected; ANIC), Mt. Mangana, Bruny I., 43°21'S 147°13'E, 4 IV 1989, Tube 8, pyrethrin knockdown *Nothofagus* J. Diggle, P. Greenslade; 1♀ (ANIC), Pirates Rd., 43°03'S 147°55'E, 16–21 III 1989, Tube 45, yellow pan trap P. Greenslade, J. Diggle, A.N.I.C COLEOPTERA Voucher No. 88-0095; 1♂ 1♀ (1♂ aedeagus dissected; ANIC), Claytons, Bathurst Harbour, 43°22'S 146°08'E, 15 III–15 IV 1991, F.I.T. #3, F.I.T. ANIC 1181 closed forest, E. Edwards, J. Berry; 1♂ (aedeagus dissected; FMNH), Mt. Field N.P. Lake Dobson Rd., Russell Falls Track, 250 m, 42°41'S 146°42'E, 25 I 1993, *Euc. regnans* forest with tree ferns, diverse understory; pyr-fogging large old *Euc*. logs,

A. Newton & M. Thayer 902; 3♂♂ 1♀ (FMNH), Mt. Field N.P. Lake Dobson Rd., Russell Falls Track, 250 m, 42°41'S 146°42'E, 25 I 1993, *Euc. regnans* forest with ferns, diverse understory; A. Newton & M. Thayer 902; 3♂♂ 3♀♀ (1♂ aedeagus dissected; FMNH), Mt. Field N.P. Lake Dobson Rd., Russell Falls Track, 250 m, 42°41'S 146°42'E, 25 I 1993, *Euc. regnans* forest with ferns, diverse understory, FMHD #93-55, berl., leaf & log litter; A. Newton & M. Thayer 902; 2♂♂ 1♀ (1♂ aedeagus dissected; FMNH), Southwest N.P., Scotts Peak Rd. (21 km S Gordon R. Rd.) Creepy Crawly National Track, 600 m, 42°50'S 146°23'E, 8–24 I 1993, *Noth. cunn*. rainforest with tree ferns (very mossy), FMHD #93-15, window trap, A. Newton & M. Thayer 904; 13♂♂ 1♀ (4♂ aedeagus dissected; 4♂♂ slide-mounted; FMNH), Murchison Highway St. Reserve, Murchison Highway at Que River Mine Rd., 680 m, 41°36'S 145°41'E, 11–27 I 1993, *Noth. cunn*. rainforest with *Atherosperma*, *Eucalyptus,* tree ferns, FMHD #93-22, window trap, A. Newton & M. Thayer 907; 5♂♂ 2♀♀ (1♂ aedeagus dissected; FMNH), Lake Chisholm Forest Res., 180 m, 41°08'S 145°04'E, 12–29 I 1993, *Euc. obliqua* forest with *Eucryphia*, *Noth. cunn*., *Blechnum* ground ferns, FMHD #93-28, window trap, A. Newton & M. Thayer 909; 1♂ 2♀♀ (FMNH), Dismal Swamp, 35.4 km WSW Smithton, 50 m, 40°57'S 144°50'E, 13–28 I 1993, *Acacia melanoxylon* swamp forest with *Noth. cunn. Euc. obliqua, Atherosperma, Melaleuca*, FMHD #93-32, carrion trap (squid), A. Newton & M. Thayer 910; 1♀ (FMNH), Ben Ridge Rd., 5.2 km E Telopea Rd., 870 m, 41°21'S 147°40'E, 16 I–1 II 1993, *Noth. cunn*. rainforest with ferns & tree ferns, FMHD #93-42, window trap, A. Newton & M. Thayer 913; 2♂♂ 2♀♀ (FMNH), Arve Loop Rd., 3.2 km NW Arve Rd., 340 m, 43°08'S, 146°46'E, 23 I 1993, *Euc. obliqua-Euc. regnans* forest with *Noth. cunn.*, FMHD #93-51, berl., leaf & log litter, A. Newton & M. Thayer 916; 1♀ (FMNH), Celery Top Pine Nature Track, ca. 11 km E Trowutta, 140 m, 41°03'S, 145°12'E, 29 I 1993, mixed forest with *Phyllocladus, Anodopetalum, Melaleuca, Acacia mucronata, Noth. cunn.*, FMHD #93-57, berl., leaf & log litter, A. Newton & M. Thayer 917; 1♂ 1♀ (UNHC), Mt. Field N.P., Lake Dobson Rd., 240 m, 30 I–5 II 1980, wet sclerophyll, window trap, A. Newton & M. Thayer; 1♂ (UNHC, slide-mounted), Mt. Field N.P., Lake Dobson Rd., 240 m, 30 I–5 II 1980, wet sclerophyll, berl., forest leaf & log litter, A. Newton & M. Thayer; 1♀ (UNHC), Mt. Field N.P., Lake Dobson Rd., 490 m, 2 II 1980, flood debris & wet moss, forest stream, A. Newton & M. Thayer; 1♂ (UNHC), 5 km SW Mt Arrowsmith Lyell Highway A10, 8–10 XII 1986, Burckhardt; 1♂ (UNHC) Hobart, 3–12 XII 1986,

D. Burckhardt; 1♂ (MVMA), F. E. Wilson Collection, COL-90209, A. Simson; 1♂ (UNHC), Tasmanian State Forest, between Huon & Weld Rivers, 60 km SW Hobart, wet sclerophyll, 43°04'S 146°40'E, 13 XII 2000, 20-30 year regrowth PRSET2, emergence trap on log, M. Yee; 1♂ (UNHC), Tasmanian State Forest, between Huon & Weld Rivers, 60 km SW Hobart, wet sclerophyll, 43°04'S 146°40'E, 13 XII 2000, 20-30 year regrowth ESET1, emergence trap on log, M. Yee; 1♂ (aedeagus dissected; ANIC), 43°22'S 146°09'E, 20 II–15 III 1991, Celery Top Islands, Bathurst Harbour, F.I.T. #2, F.I.T. ANIC 1160, closed forest, M. Horak, P. McQuillan; 3♂♂ 2♀♀ (1♂ aedeagus dissected; 1♂ slide-mounted; UNHC), Swan, Strahan, 19 I 2010, Sifted ground moss; 1♂ (UNHC), 35 km SW Smithton, Christmas Hills, 27 II 1977, FMHD #77-177, *N. cunninghamii* litter around log, J. Kethley; 1♂ (UNHC), Lower Gordon R., 42°31'S 145°45'E, 42°32'S, 145°47'E, I 1978, Howard, Hill…, H.E.C. Survey 3R 600, litter; 1♂ (UNHC), St. Columba Falls, 315 m, 12–14 II 1980, mixed forest with *Nothofagus cunninghamii* and tree ferns, berl. forest leaf & log litter, A. Newton & M. Thayer. **Victoria:** 1♂ 1♀ (UNHC), Mt. Buffalo N.P., Eurobin Creek, 1,300 m, 24 IV 1978, Eucalyptus litter, S. & J. Peck; 1♀ (UNHC), Mt. Buffalo N.P., Eurobin Creek, 1,300 m, 24 IV 1978, debris under bark, S. & J. Peck; 6♂♂ 1♀ (UNHC), Otway N.P., Maits Rest, 260 m, 38°45'S 143°33'E, 25 I–8 II 1987, wet scleroph, -*Noth*. *cunn*., FMHD #87-206, flight intercept (window) trap, A. Newton & M. Thayer 807; 3♂♂ 3♀♀ (UNHC), Otway N.P., Binn Rd., 4.3 km N Cape Horn, 390 m, 38°43'S 143°35'E, 25 I–8 II 1987, wet sclerophyll forest, FMHD #87-210, flight intercept (window) trap, A. Newton & M. Thayer 808; 2♂♂ (UNHC), Otway Range, Haines Jct., 1.9 km W on Turtons Track, 525 m, 38°39'S 143°42'E, 25 I–8 II 1987, wet sclerophyll forest, FMHD #87-213, flight intercept (window) trap, A. Newton & M. Thayer 809; 1♀ (UNHC), Otway Range, Haines Jct., 1.9 km W on Turtons Track, 525 m, 38°39'S 143°42'E, 25 I–8 II 1987, wet sclerophyll forest, FMHD #87-215, berl., leaf & log litter, forest floor, A. Newton & M. Thayer 809; 2♂♂ (1♂ aedeagus dissected; UNHC), Tanjil Bren, 6.1 km ESE, 590 m, 37°50'S 146°12'E, 29 I–10 II 1987, wet sclerophyll forest, FMHD #87-244, flight intercept (window) trap, A. Newton & M. Thayer 818; 2♂♂ (1♂ aedeagus dissected; UNHC), Keppel Falls Scen. Res., ENE Marysville, Myrtle Loop Trail, 780 m, 16–28 II 1993, cool temperate rainforest, FIT, A. Newton & M. Thayer; 1♀ (UNHC), Baw Baw Alpine Res. nr. Newlynes Mill, 1,035 m, 26 II 1993, fogging *Eucalypt.*

regnans log, A. Newton & M. Thayer; 1♀ (MVMA), Ferntree Gully, 6 VII 1924, C. Oke; 1♂ (MVMA), Black's Spur, I 1934, COL-90211, C. Oke; 1♂ (MVMA), Balook V, COL-90210, C. Oke.

Australosagola rugicornis **(Oke, 1932), comb. nov.**

Other material examined (*n* = 23; 9♂♂, 14♀♀)**. Australia: Victoria:** 2♂♂ 1♀ (1♂ aedeagus dissected; 1♀ slide-mounted; UNHC), Baw Baw Alpine Res., 1.2 km NE Neulynes Mill, wet scleroph. -*Noth. cunn.*, 1,145 m, 37°51'S 146°15'E, 29 I 1987, A. Newton & M. Thayer 816 FMHD #87-240 berl., leaf & log litter, forest; 1♂ 3♀♀ (1♂ slide-mounted; UNHC), Mt. Donna Buang, N Warburton, wet scleroph. -*Noth. cunn.*, 1,200 m, 37°43'S 145°41'E, 26 I–11 II 1987, FMHD #87-216 flight intercept (window) trap, A. Newton & M. Thayer 810; 3♀♀ (1♀ slide-mounted; UNHC), Mt. Donna Buang, N Warburton, *Noth. cunn.* -wet scleroph., 1,200 m, 37°43'S 145°41'E, 26 I–11 II 1987, FMHD #87-219 flight intercept (window) trap, A. Newton & M. Thayer 811; 2♂♂ (1♂ dissected; 1♂ aedeagus dissected; UNHC), Mt. Donna Buang, N Warburton, *Noth. cunn.* -wet sclerophyll, 1,200 m, 37°43'S 145°41'E, 6 II 1987, pyrethrin-fogging old fungusy *Eucalyptus* logs, A. Newton & M. Thayer 811; 1♂ 1♀ (1♂ slide-mounted; UNHC), Baw Baw Alpine Res., 1 km WNW Alpine Village, *Euc. pauciflora* woodland, 1,420 m, 37°50'S 146°16'E, 29 I 1987, FMHD #87-243 berl., leaf & log litter, forest floor A. Newton & M. Thayer 817; 2♀♀ (UNHC), Mt. Donna Buang, *Eucalyptus-Nothofagus* forest, 1,200 m, 11–17 I 1980, window trap, A. Newton & M. Thayer 550; 1♂ (slide-mounted; UNHC), Mt. Donna Buang, 37°43'S 145°41'E, 19 I 1987 under bark rotten logs, V. Lawerence & Weir; 1♀ (UNHC), Acheron Gap, NE Warburton, *Noth. cunn. -Euc. regnans*, 750 m, 37°41'S 145°44'E, 27 I 1987, FMHD #87-228 berl., leaf & log litter, forest floor (north side of gap), A. Newton & M. Thayer 813; 1♀ (UNHC), Acheron Gap, NE Warburton, *Noth. cunn. -Euc. regnans*, 750 m, 37°41'S 145°44'E, 4 II 1987, FMHD #87-231 berl., sifted rotting wood of *Noth. cunn.* and *Eucalyptus* logs, A. Newton & M. Thayer 813; 1♂ (UNHC), Cumberland Sc. Res. SW Cambarville, Cora Lynn Falls, *Euc. regnans-Noth. cunn.*, 880 m, 37°34'S 145°53'E, 5 II 1987, FMHD #87-257 berl., leaf & log litter, forest floor, A. Newton & M. Thayer 824; 1♀ (UNHC), Cumberland Creek 13 km ESE Marysville, 18 I 1978, berl. leaf litter, J. Lawrence & T.

Weir; 1♀ (FMNH), Mt. Margaret Rd. at Ghost Point, NNE Marysville, open *Euc. delegatensis* forest, 1,070 m, 37°27'S 145°47'E, FMHD #93-107, berl., leaf & log litter, 16 II 1993, A. Newton & M. Thayer 933. **New South Wales:** 1♂ (slide-mounted; FMNH), Kosciusko N.P. 4.1 km W Dead Horse Gap, *Euc*. *pauciflora* woodland, 1,500 m, 36°32'S 148°13'E, 19 XII 1986–14 II 1987, FMHD #86-645, flight intercept (window) trap, A. Newton & M. Thayer 766.

Australosagola helenae **(Oke, 1925), comb. nov.**

Other material examined (*n* = 30; 17♂♂, 13♀♀)**. Australia: Australian Capital Territory:** 1♂ (aedeagus dissected; UNHC), Brindabella Range 2 km N. Mt. Aggie, 1,400 m, 19 XI 1991, Berlese dead wood include red rotten logs, M.A. & L.L. Ivie & J.F. Lawrence; 1♂ 1♀ (aedeagus dissected; ANIC), Piccadilly Circus, 1,240 m, 35°22'S 148°48'E, V 1984, flight intercept window/trough trap, J. Lawrence, T. Weir M-L. Johnson, coll.; 1♂ (aedeagus dissected; ANIC), Blundells Ck. 3 km E of Piccadilly Circus, 850 m, 35°22'S 148°50'E, V 1985, flight intercept window/trough trap, Weir, Lawrence, Johnson. **New South Wales:** 1♂ (aedeagus dissected; FMNH), Kosciusko N.P. 4.1 km W Dead Horse Gap, *Euc. pauciflora* woodland, 1,500 m, 36°32'S 148°13'E, 19 XII 1986–14 II 1987, FMHD #86-645, flight intercept (window) trap, A. Newton & M. Thayer 766; 1♂ (aedeagus dissected; UNHC), Mt. Brindabella, Piccadilly Circus, 21 XII 1986, Burckhardt; 1♂ (slide-mounted; UNHC), Mt. Brindabella, Lee’s Spring, 21 XII 1986, Burckhardt; 1♂ (aedeagus dissected; SAMA), Dividing Rge., V, Blackb’s Coll.. **Victoria:** 1♀ (UNHC), Warburton, Acheron Way, *Euc. -Nothofagus* 2nd-gr. for., 495 m, 11–16 I 1980, berl. forest leaf & log litter. A. Newton, M. Thayer; 1♂ (mounted in micro vial; MVMA), Alps, V., C. Oke; 1♂ (UNHC), Mt. Worth N.P., Trevorrows Mill, wet sclerophyll forest, 300 m, 38°17'S 146°00'E, 7 II 1987, FMHD #87-237 berl., leaf & log litter, forest floor, A. Newton & M. Thayer 816; 1♂ (UNHC), Mt. Dandenong, Belgrave, Zwick; 1♂ (slide-mounted; UNHC), Wilson’s Prom. N.P. Lilly Pilly Trail, 14 V 1978, leaf & log litter, S. & J. Peck; 1♀ (UNHC), Wilson’s Prom. N.P. Lilly Pilly Trail, 15 V 1978, S. & J. Peck; 1♂ 2♀♀ (UNHC), Tanjil Bren, 6.1 km ESE, wet sclerophyll forest, 590 m, 37°50'S 146°12'E, 29 I 1987, FMHD #87-246 berl., leaf & log litter, forest floor, A. Newton & M. Thayer 818; 2♂♂ (UNHC), Tanjil

Bren, 6.1 km ESE, wet sclerophyll forest, 590 m, 37°50'S 146°12'E, 29 I–10 II 1987, FMHD #87-244 flight intercept (window) trap, A. Newton & M. Thayer 818; 2♀♀ (FMNH), Mt. Margaret Rd. at Ghost Point, NNE Marysville, open *Euc. delegatensis* forest, 1,070 m, 37°27'S 145°47'E, 16 II 1993, FMHD #93-107, berl., leaf & log litter, A. Newton & M. Thayer 933; 1♀ (UNHC), Cook N.P. 25 km S of Cann River, *Eucalyptus* litter and bark, 24 V 1978 S. & J. Peck; 1♂ (UNHC), Acheron Gap, NE Warburton, *Noth*. *cunn. -Euc. regnans*, 750 m, 37°41'S 145°44'E, 27 I 1987, pyrethrin-fogging old *Nothofagus cunninghamii* logs, A. Newton & M. Thayer 813; 1♂ (UNHC), Acheron Gap, NE Warburton, *Noth*. *cunn. -Euc. regnans*, 750 m, 37°41'S 145°44'E, 9 II 1987, pyrethrin-fogging old fungusy logs, A. Newton & M. Thayer 813; 1♀ (UNHC), Olinda Falls, nr. Olinda, *Eucalyptus*, c350 m, 15 I 1980, berl. forest leaf & log litter, A. Newton, M. Thayer; 1♂ 4♀♀ (1♂ aedeagus dissected; 1♀ slide-mounted; UNHC), Mt. Buffalo N.P. Eurobin Ck., 1,300 m, 24 IV 1978, *Eucalyptus* litter, S. & J. Peck.
